# An incoherent feed-forward loop switches the Arabidopsis clock rapidly between two hysteretic states

**DOI:** 10.1038/s41598-018-32030-z

**Published:** 2018-09-17

**Authors:** Ignasius Joanito, Jhih-Wei Chu, Shu-Hsing Wu, Chao-Ping Hsu

**Affiliations:** 1 Institute of Chemistry, Academia Sinica, Taipei, 11529 Taiwan; 2 Bioinformatics Program, Taiwan International Graduate Program, Academia Sinica, Taipei, 115 Taiwan; 3 Institute of Bioinformatics and System Biology, National Chiao Tung University, Hsinchu, 300 Taiwan; 4 Institute of Plant and Microbial Biology, Academia Sinica, Taipei, 11529 Taiwan; 5 Department of Biological Science and Technology, National Chiao Tung University, Hsinchu, 300 Taiwan; 6 Genome and Systems Biology Degree Program, National Taiwan University, Taipei, 106 Taiwan

## Abstract

In higher plants (e.g., *Arabidopsis thaliana*), the core structure of the circadian clock is mostly governed by a repression process with very few direct activators. With a series of simplified models, we studied the underlying mechanism and found that the *Arabidopsis* clock consists of type-2 incoherent feed-forward loops (IFFLs), one of them creating a pulse-like expression in *PRR9/7*. The double-negative feedback loop between *CCA1/LHY* and *PRR5/TOC1* generates a bistable, hysteretic behavior in the *Arabidopsis* circadian clock. We found that the IFFL involving *PRR9/7* breaks the bistability and moves the system forward with a rapid pulse in the daytime, and the evening complex (*EC*) breaks it in the evening. With this illustration, we can intuitively explain the behavior of the clock under mutant conditions. Thus, our results provide new insights into the underlying network structures of the *Arabidopsis* core oscillator.

## Introduction

The circadian clock is an endogenous time-keeping mechanism in cells and organisms that anticipates daily changes in the environment^1–4^. It controls the daily rhythms of many biological processes such as gene expression, biochemical pathways, metabolism, physiology, and memory formation^5–7^. Disruption of the clock has been associated with many disadvantageous traits such as reduced plant growth and fitness^8^, human cancer^9^, metabolic diseases^10^, and also aging in mice^11^. Despite the low homology and vast differences in complexity among different species, most circadian clocks still share a common network structure at the core level^12^. Therefore, the study of circadian clock dynamics in relation to its network structure has become increasingly important.

In many eukaryotes, the molecular mechanisms of the circadian clock are rooted in coupled transcription–translation feedback loops consisting of direct activators and repressors that form coupled negative and positive feedback loops^2–4^. Previous study showed that a single negative feedback loop can generate sustained oscillations^13^. However, a positive feedback loop has been found responsible for generating bistability in many systems^14–17^. A combination of both positive and negative feedback loops can lead to robust oscillation with desirable properties such as noise resistance^18^ and tunable frequency^19^. Thus, the coupled positive and negative feedback loops could be advantageous for the circadian clock such that it is retained in many organisms throughout evolution.

In contrast to the direct activation and repression process in fungi, flies, and mammals, the current known core oscillator of the *Arabidopsis thaliana* circadian clock has few direct activators. Although several activators, such as *LIGHT-REGULATED WD1* (*LWD1*)^20,21^ and *REVEILLE8* (*RVE8*)^22,23^, have been found recently, the core oscillator is still predominantly composed of repression regulations^24,25^. More than 20 clock or clock-associated genes, which act at distinct times throughout the day and night cycle, have been identified in *Arabidopsis*^24^. For instance, in the early morning, transcripts of *CIRCADIAN CLOCK-ASSOCIATED1* (*CCA1*) and *LATE ELONGATED HYPOCOTYL* (*LHY*) are accumulated and repress many nighttime genes, such as *TIMING OF CAB EXPRESSION 1* (*TOC1*), *PSEUDO-RESPONSE REGULATOR 5* (*PRR5*), *LUX ARRHYTHMO* (*LUX*), and *EARLY FLOWERING 4* (*ELF4*)^26–31^. Furthermore, these *CCA1* and *LHY* genes promote the expression of several daytime/noon-phased genes such as *PRR9* and *PRR7*, which in turn inhibits *CCA1* and *LHY* expression^29,32^. Recently, *LUX*, *ELF4*, and *ELF3* were found to form a complex in the middle of the night, defined as the evening complex (*EC*)^33^. This *EC* has also been proposed to play a crucial role in inhibiting *PRR* genes^34–36^ and tracking seasonal change in both photoperiod and ambient temperature^37,38^.

Among these dense repression processes, we can find another network motif called the incoherent feed-forward loop (IFFL), which has not been widely discussed in the clock systems. In the mammalian clock, IFFLs are the most frequently occurring potential network motifs^39^. IFFLs have also been shown to play many important roles in cells, such as speeding up the output response and generating pulse-like dynamics^40,41^, facilitating biochemical adaptation^42^, providing a fold-change detection that buffers stochastic variation^43,44^, and promoting temperature robustness^45^. However, the roles of these IFFLs in the oscillating system, such as the circadian clock, are still elusive.

Therefore, in this study, we built a series of simplified mathematical models for qualitative insight into the *Arabidopsis* clock system. With this insight, we aim to learn from the underlying network structures of the core oscillator and understand their advantages in the clock systems. The *Arabidopsis* clock, like other eukaryotic clocks, possesses a negative feedback loop (similar to that of the repressilator^46^) and a positive feedback loop (double negative feedback) in the core oscillator. It also contains IFFLs. In the following, we show that one of the positive feedback loops, the one between *CCA1/LHY* and *PRR5/TOC1*, features a bistable, hysteretic behavior in the *Arabidopsis* circadian clock. While the negative feedback loop pushes the oscillation forward, the IFFL generates a pulse-like expression in *PRR9/7*, forming rapid switches between the two states. With this simple illustration, we provide an intuitive explanation for why under the *cca1;lhy*, *toc1*, or *prr5* mutant condition the clock moves faster, while under the *prr9* and *prr7* mutant condition, it moves slower. Moreover, we also found similar dynamics in the more complex and detailed models published previously^47,48^. Thus, our work offers intuitive understanding for the clock mechanism in plants.

## Results

### The direct inhibition and indirect activation of *CCA1/LHY* to *PRR9/7* are important for robustness and correct dynamics of clock system

We first simplified the *Arabidopsis* clock to obtain the “core” structure for further study. Considering the large number of clock genes and high level of functional similarity among them, we merged similar genes into a single variable to reduce the number of equations and unknown parameters (Supplementary Information). This approach has been commonly used in many mathematical models such as in describing the expression of *CCA1* and *LHY* (*CCA1/LHY*)^47,49–51^, *PRR9* and *PRR7* (*PRR9/7*)^51,52^, as well as *ELF4* and *LUX*^47,48,51^. Next, to increase the general applicability of the conclusions drawn from the modeling, we randomly generated all the parameter values in the model and selected parameter sets that generated sustained oscillations, with the *lwd1/*2 mutant correctly reproducing a shorter period (>3 hr) and lower amplitude (>50%) in both *PRR9/7* and *CCA1/LHY* (as shown in experimental results^20^). Furthermore, all of our simulations were performed under constant light (LL) because we focused on insights into the intrinsic dynamic properties of the clock system (See Methods for further details).

#### Qualitative analysis led to revision of the previous simplified model

**Figure 1:**
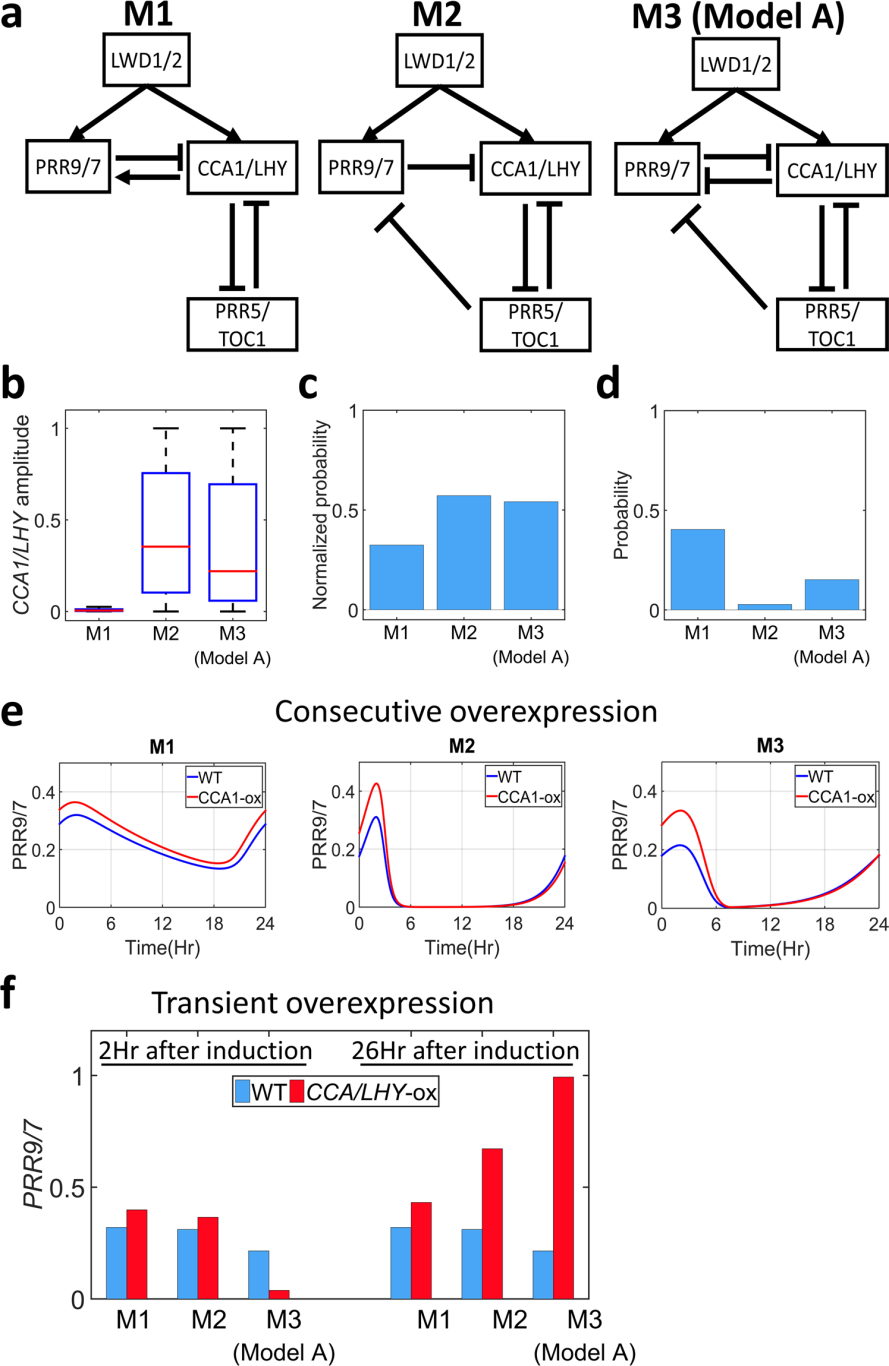
Comparison of three different models. (**a**) Schematic representation of the tested models. (**b**) Box plot representing the oscillation amplitude of *CCA1/LHY* genes for all parameter sets. Red lines indicate the median, and box edges indicate the 25^th^ (Q1) and 75^th^ (Q3) percentiles. Whiskers are plotted at 1.5*(Q3-Q1). (**c**) The averaged hitting rate of each parameters in the three models for regular oscillation and correct *lwd1/2* mutant dynamics. (**d**) The probability that the obtained parameter sets showed correct period changes in the genetic perturbation test results (*cca1/lhy*, *prr9/7*, *prr5/toc1*) for each model. (**e**,**f**) The expression of *PRR9/7* gene under wild-type (blue) or *CCA1/LHY* overexpression (red) with a consecutive promoter (E) or transient induction examined at 2 or 26 hr after induction (F).

We started our study with model M1 (Fig. 1a, left panel), which has been used to show that *LWD1/2*, together with TCP proteins, were activators of *CCA1*^21^. However, we found that *CCA1/LHY* were always under tight repression in most parameter sets, thereby resulting in very low amplitude (Fig. 1b). This tight repression is less likely to occur because it can be broken easily with a slight perturbation, or noise^53^. Therefore, we revisited the model structure and sought another plausible interaction to be included.

Previously, positive effect of *CCA1* and *LHY* to *PRR9* and *PRR7* was suggested to occur from direct or indirect processes^54^. Later studies showed that *PRR5* and *TOC1* could bind to *PRR9* and *PRR7* promoters and reduce their expression^55,56^. Together with *CCA1* and *LHY* inhibition of *PRR5* and *TOC1*, it forms an indirect activation of *CCA1*/*LHY* to *PRR9*/*PRR7*. Thus, we removed the direct interaction of *CCA1/LHY* to *PRR9/7* and added a direct inhibition of *TOC1/PRR5* to *PRR9/7*, forming model M2 (Fig. 1a, Middle). Despite ChiP-qPCR assay has shown that *LWD1/2* can bind on both *PRR5* and *TOC1* promoter regions^20^, we found a substantial decrease in the performance in model M1 when we added this direct activation (Supplementary Fig. S1). Since there is no additional evidence of this interaction, we were reluctant to add this interaction into our model M2.

Of note, instead of being activators, recent data showed that the expression of *PRR9* and *PRR7* were reduced soon after the induction of *LHY* or *CCA1*^57,58^. However, the positive effect of *LHY* on both *PRR9* and *PRR7* could still be observed in a longer time window^57^. These findings are intriguing because they imply a fast inhibition but a slower activation of *LHY* to *PRR7/9*. Therefore, we included another model, M3 (model A), to accommodate these findings and studied the importance of this direct inhibition (Fig. 1a, Right).

#### Indirect activation of CCA1/LHY to PRR9/7 improved robustness of the clock systems

To test the effect of changing the direct into indirect activation, we compared the performance of the M1 and M2 models. The expression of *CCA1/LHY* in model M2 was no longer repressed tightly, as seen in the amplitudes that distribute normally (Fig. 1b). Moreover, the normalized hitting rate of each parameter in model M2 was much higher (almost twice) than in model M1 (Fig. 1c, Table S1). A tight repression requires a close match in the parameter space and thus a much lower hitting rate in model building. Such increase in parameter hitting rates also implies an increase in the robustness of the system because it is able to tolerate more parameter combinations. However, model M2 performed poorly in replicating the genetic perturbation test (Fig. 1d). Most parameter sets in model M2 failed to reproduce the longer periods under the *prr9/7* mutant condition as experimentally observed (Supplementary Fig. S2A). This result indicated that the indirect activation of *CCA1/LHY* to *PRR9/7* improved the robustness of the system although the structures of model M2 were not sufficient in representing the overall behavior of the clock system.

#### Direct CCA1/LHY inhibition of PRR9/7 is important in replicating the correct dynamics of Arabidopsis clock system

Next, we tested whether adding the *CCA1/LHY* direct inhibition of *PRR9/7*^57,58^ could improve the model performance by comparing the M3 and M2 models. Our simulation results showed that the amplitude of *CCA1/LHY* was normally distributed in model M3 (Fig. 1b), and the normalized hitting rate was comparable for both models (Fig. 1c). However, we found a noticeable improvement in the performance of the genetic perturbation test, which was 5-fold higher for M3 than M2 (Fig. 1d). This improvement was mostly due to better performance of the *prr9/7* mutant condition in model M3 (Supplementary Fig. S2A). Thus, the direct *CCA1/LHY* inhibition of *PRR9/7* may be important in reproducing the correct *prr9/7* mutant dynamics.

We further tested the performance of our models by replicating different experiments for *CCA1/LHY* overexpression effects on *PRR9/7*. First, we consecutively overexpressed *CCA1/LHY* in all models and monitored *PRR9/7* expression (Method). *PRR9/7* expression was elevated in all models, which agrees with previous experimental results (Fig. 1e and Supplementary Fig. S3)^32^. In model M3, the parameter distribution indicates a relatively high threshold for *CCA1/LHY* direct inhibition of *PRR9/7* as compared with the indirect activation through *PRR5/TOC1* (Supplementary Fig. S2B,C). As a result, the indirect activation dominated the overall expression. We also used transient overexpression of *CCA1/LHY* followed by measuring the expression of *PRR9/7* at 2 and 26 hr after the induction (Method). As expected, most parameter sets in all models were able to replicate the elevated *PRR9/7* expression 26 hr after the induction, but only model M3 could replicate the reduction of *PRR9/7* at 2 hr after the induction because of the direct *CCA1/LHY* inhibition of *PRR9/7* (Fig. 1f and Supplementary Fig. S4). Therefore, these results demonstrated that the direct *CCA1/LHY* inhibition and indirect *CCA1/LHY* activation of *PRR9/7* are important for the system dynamics. Moreover, using this representation, model M3 can qualitatively represent and explain the clock dynamics shown in the experimental results. Hence, in this study, we proceeded with M3, which will be called as model A onward, to gain insights into the *Arabidopsis* clock.

### Dynamics of the network motifs in the *Arabidopsis* core clock

**Figure 2:**
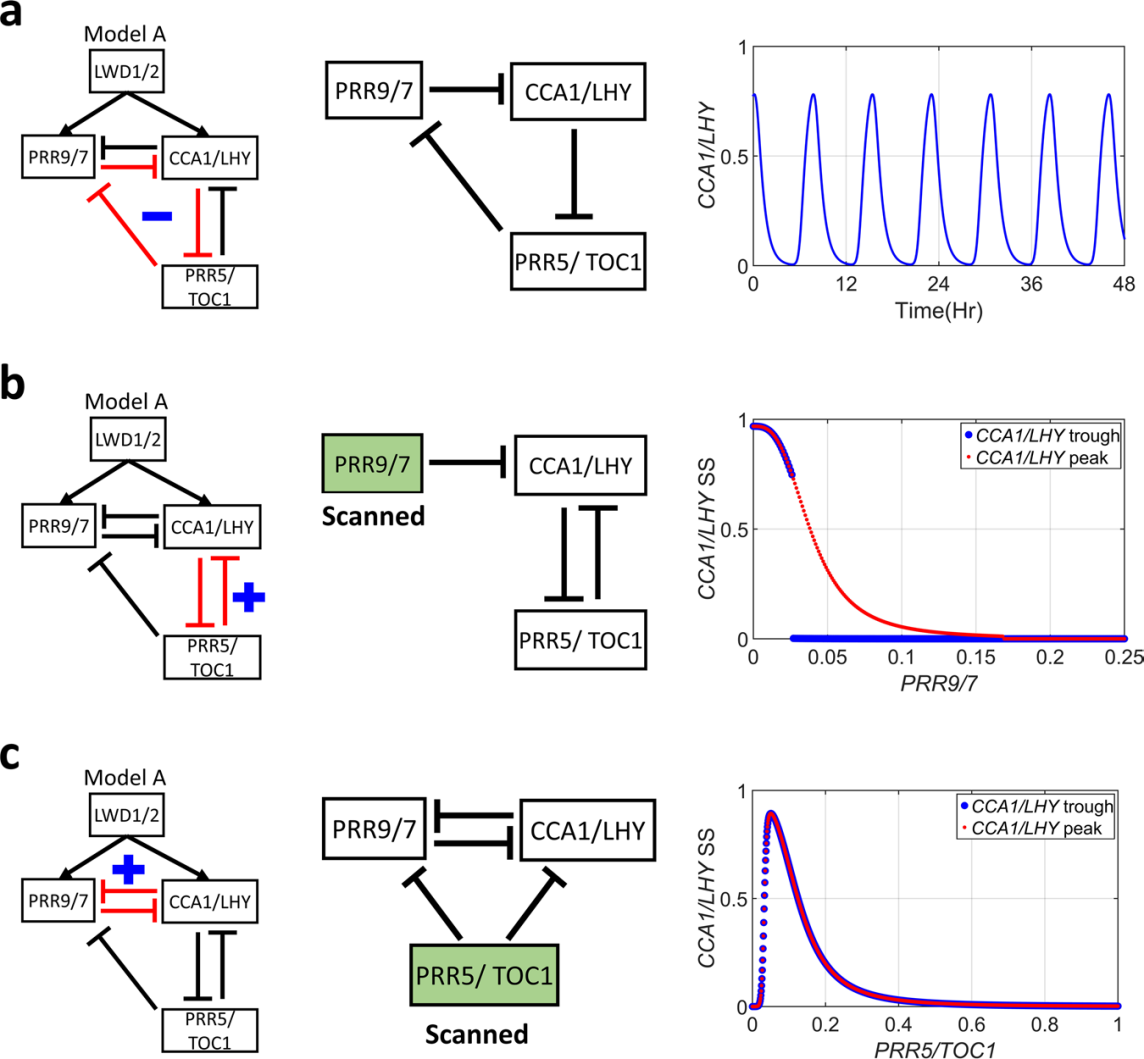
The negative and positive feedback loops in model A. The motif studied as highlighted in red. The sub-network tested. Genes in the green box (panel b and c) are fixed at a value in the propagation for steady states. The level of genes in the green boxes were then scanned. The dynamics of tested sub-networks. (**a**) *CCA1/LHY* concentration under constant light condition. (**b**,**c**) Dots represent the steady-state (SS) value of *CCA1/LHY* concentration starting from a low level of *CCA1/LHY* (blue dots) or a high level of *CCA1/LHY* (red dots), plotted as a function of the fixed value of *PRR9/7* (**b**) or *PRR5/TOC1* (**c**).

**Figure 3:**
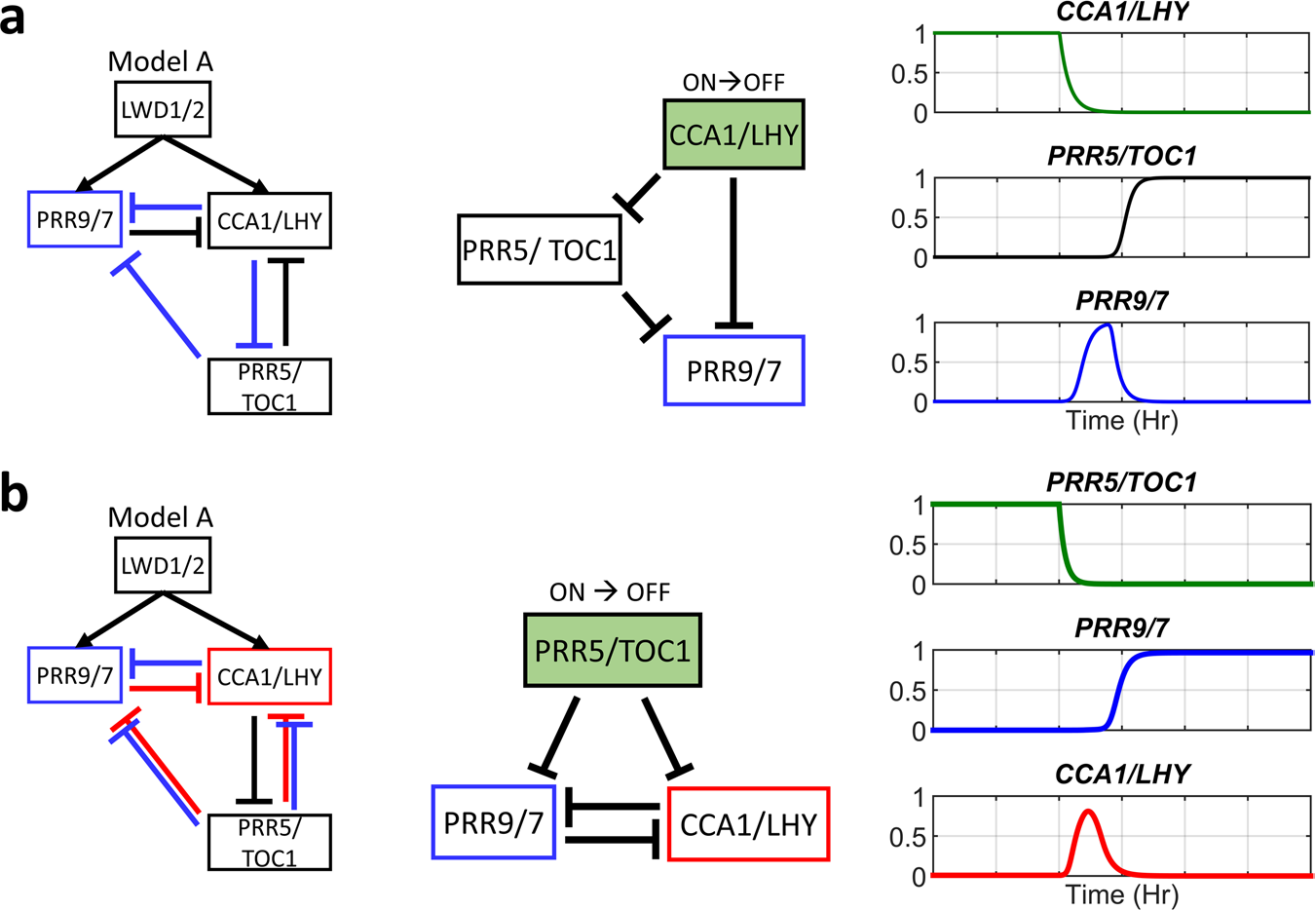
Type-2 incoherent feed-forward loop (IFFL-2) in model A. The motif studied as highlighted in blue/red. The sub-network that would be tested. The dynamics of genes in the green boxes are given as input functions mimicking the turned-off dynamics during the simulation. The behavior of the tested sub-network. The simulation was started from an initially high level of *CCA1/LHY* (**a**) or initially high level of *PRR5/TOC1* (**b**).

Many studies have indicated that transcriptional networks contain a small set of recurring regulation patterns as network motifs^40,59–61^. We found that model A has three different network motifs: a negative feedback loop, positive feedback loops (double-negative feedback), and IFFLs (Figs 2 and 3). Thus, we further analyzed their effect on clock dynamics.

#### Negative feedback drives the oscillation

As shown in Fig. 2a (left), model A contains a negative feedback loop, or the repressilator. A repressilator is a three-inhibitor feedback loop that was originally constructed as a synthetic circuit and capable of generating oscillations in *Escherichia coli*^46^. To test the roles of this repressilator, the network was reduced by removing the *CCA1/LHY* inhibition of *PRR9/7* and *PRR5/TOC1* inhibition of *CCA1/LHY* (Fig. 2a middle). We found that the partial network still oscillated but with altered period (Fig. 2a right). Furthermore, this property was observed for 88.37% of the parameter sets obtained for model A. Because of no other negative feedback loop in the system nor other oscillation-generating parts can be derived in model A, this result indicates that the repressilator drives the oscillation.

#### Double-negative feedback between CCA1/LHY and PRR5/TOC1 generates a hysteresis

Many studies have shown that a positive feedback loop can promote a bistable, hysteretic behavior^14–17^. Therefore, we tested the two positive feedback loops in model A for bistability (Fig. 2b,c). We first examined the *CCA1/LHY* and *PRR5/TOC1* double-negative feedback by keeping a constant *PRR9/7* as an input, at various concentrations (Fig. 2b middle). The steady-state *CCA1/LHY* concentration was determined as a function of *PRR9/7* (See Methods). The result showed that starting from an initially high *CCA1/LHY* concentration (red dots), the *PRR9/7* concentration must exceed 0.16 to completely repress *CCA1/LHY*. However, *PRR9/7* could repress *CCA1/LHY* expression at 0.03 when the system started from an initially low concentration of *CCA1/LHY* (blue dots) (Fig. 2b right). At the intermediate concentration, for *PRR9/7* (0.03-0.16), *CCA1/LHY* had two stable steady states depending on the initial state, a clear characteristic of bistability (Fig. 2b right). Furthermore, this bistability occurred for 93.24% of the selected parameter sets. The highly enriched bistable parameters sets suggest that bistability helps reproduce the correct behavior of the clock system.

Next, we also performed a similar analysis for the double-negative feedback between *CCA1/LHY* and *PRR9/7* (Fig. 2c) by varying the concentration of *TOC1/PRR5*. We could not find any bistability in *CCA1/LHY* and *PRR9/7* in all selected parameter sets obtained for model A (Fig. 2c right). In contrast to the previous positive feedback loop, *TOC1/PRR5* inhibited both *PRR9/7* and *CCA1/LHY* in the *CCA1/LHY-PRR9/7* feedback loop. Consequently, the increase in *TOC1/PRR5* expression also reduced the strength of the positive feedback, so the bistability could not be observed in this sub-network. Therefore, our results suggest that only the *CCA1/LHY* and *PRR5/TOC1* double-negative feedback loop is likely to confer bistable, hysteretic behavior in the *Arabidopsis* clock system.

These results suggest that one of the positive feedback loops in the *Arabidopsis* clock creates a toggle switch, whereas the negative feedback, through *PRR9/7*, breaks the bistability in the daytime. This bistable and switching scheme can be used to interpret the shorter or longer periods in several mutants. For instance, the mutation *cca1*, *lhy*, *prr5* or *toc1* weakens the positive feedback, which reduces the bistability. This weakening in the bistability allows the system to move faster from one state to another; thus, we can observe the shortening period in the clock system. However, a mutation in *PRR9* and *PRR7* reduces the ability of the system to break the bistability, and thus it takes longer to switch the phases, leading to a longer oscillating time, as seen in previous experimental results^26,62,63^.

#### IFFL creates pulse-like expression of PRR9/7 and CCA1/LHY

We note that the *CCA1/LHY* direct inhibition and indirect activation of *PRR9/7* forms another network motif called the IFFL, more specifically, type-2 IFFL (IFFL-2) (Fig. 3a left)^40^. Previous studies have shown that an IFFL-2 could accelerate the response time and generate pulse-like expression of the target gene^40^. Therefore, we tested the roles of IFFL-2 in model A by turning *CCA1/LHY* off (Fig. 3a Middle, Methods). We found that *PRR9/7* had a pulse-like expression when *CCA1/LHY* expression was suddenly decreased (Fig. 3a right), and a similar result was observed for all selected parameter sets obtained in this study. Furthermore, we performed a similar analysis for the other two IFFLs found in model A (Fig. 3b). However, for 88.16% of the parameter sets, only *CCA1/LHY* exhibited a pulse-like expression with a sudden decrease in expression of *PRR5/TOC1* (Fig. 3b right) but not *PRR9/7*. For generating a pulse in IFFL-2, the downstream gene needs to have a sufficient delay time to accumulate before the intermediate gene starts to inhibit the expression (Supplementary Fig. S5A). For selected parameter sets of model A, the *PRR5/TOC1* inhibition of *PRR9/7* must be stronger than the *PRR5/TOC1* inhibition of *CCA1/LHY*; otherwise, it would disrupt the oscillation (Supplementary Fig. S5B). As a result, only *CCA1/LHY* had sufficient time to accumulate before *PRR9/7* started to inhibit its expression. Thus, our results suggest that the IFFL-2 is able to generate pulse-like expression in *PRR9/7* and *CCA1/LHY*.

#### IFFL rapidly switches PRR9/7, which is important for clock dynamics

As discussed previously, adding the *CCA1/LHY* direct inhibition of *PRR9/7* improves the model’s performance (Fig. 1). We hypothesized that this improvement might occur due to the addition of IFFL-2 from *CCA1/LHY* to *PRR9/7*, which is present in model A but lacking in the previous tested model (M2 in Fig. 1a).

**Figure 4:**
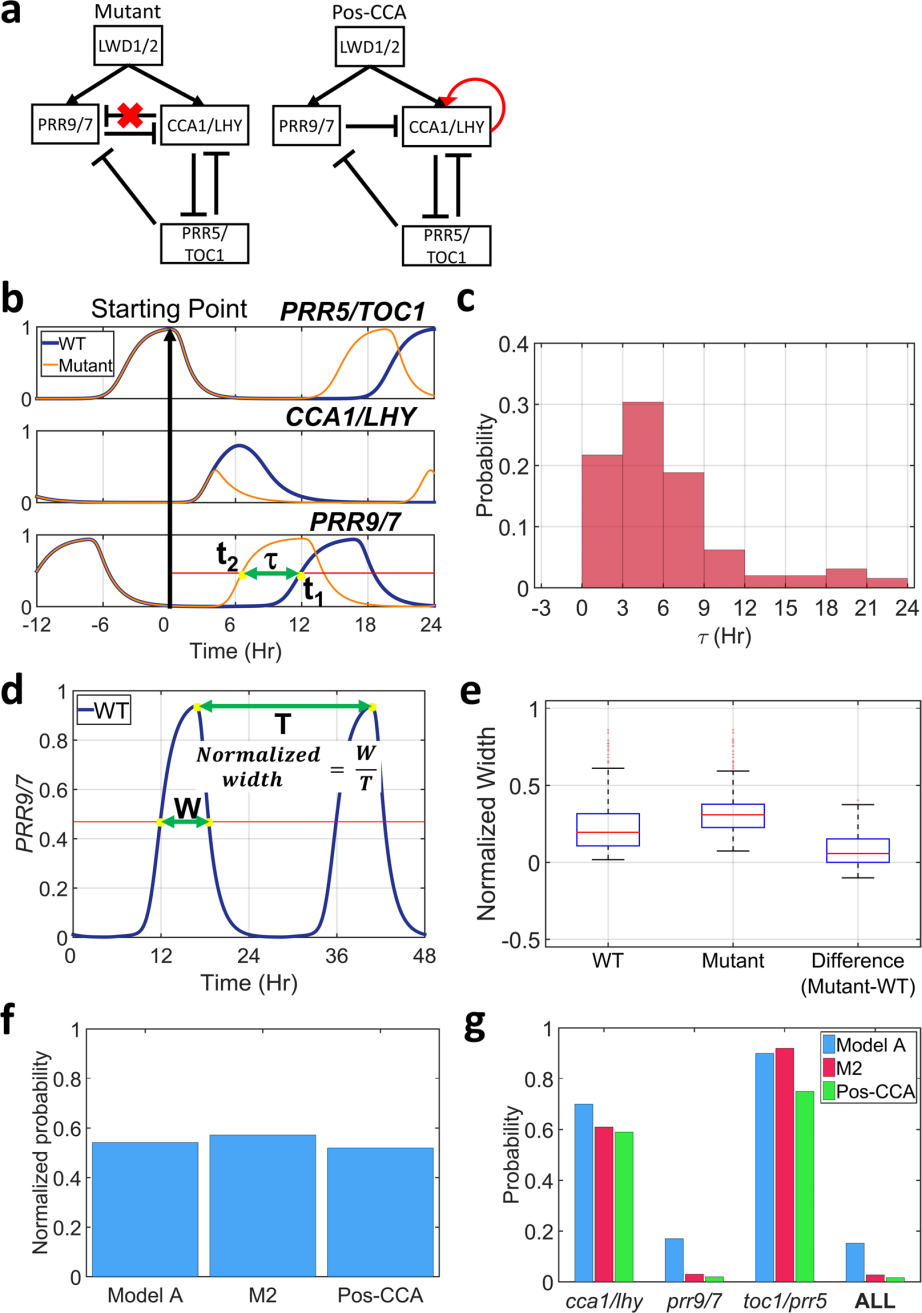
The IFFL-2 from *CCA1/LHY* to *PRR9/7* is important for clock dynamics. (**a**) Schematic representation of the tested model. (**b**) The definition of the delay time. t_1_ and t_2_ represent the time when *PRR9/7* expression reached 50% of peak expression in the wild type (WT) and mutant, respectively. The delay time (τ) was calculated by subtracting t_2_ from t_1_. (**c**) The probability distribution of the delay time (τ) for most selected parameter sets. Parameter sets that did not show any delay or advance expression were excluded (18.18%). (**d**) Illustration of how normalized width was calculated. “W” represents the full width at half maximum (FHMW), and “T” represents the oscillation periods. (**e**) Box plot representing the normalized width of *PRR9/7* gene for all parameter sets. Red line indicates the median, and box edges indicate the 25^th^ (Q1) and 75^th^ (Q3) percentiles. The whiskers are defined as 1.5*(Q3-Q1). (**f**) The averaged hitting rates of each parameters showing regular oscillation and correct *lwd1/2* mutant dynamics. (**g**) The probability that the obtained parameter sets showed correct period changes in the genetic perturbation test results (*cca1/lhy*, *prr9/7*, *prr5/toc1*) for each model.

To test this idea, we first analyzed whether IFFL-2 would delay *PRR9/7* expression due to direct inhibition of *CCA1/LHY*. The *PRR9/7* expression time was calculated in a case with lack of *CCA1/LHY* inhibition of *PRR9/7* (mutant), as compared to that in the wild type, starting from a high level of *PRR5/TOC1* (Fig. 4a,b, and Method). For 81.82% of selected parameter sets, the *PRR9/7* expression was delayed due to *CCA1/LHY* inhibition of *PRR9/7*, with the mean value of about 5 H (Fig. 4b,c). Consequently, this delay gave *CCA1/LHY* a longer time to accumulate and thus create a larger amplitude (Supplementary Fig. S6). However, for the other 18.18% of the parameter sets, the *PRR9/7* expression was not delayed because of the inactive threshold of *CCA1/LHY* inhibition of *PRR9/7* because of random sampling. In those cases, the *CCA1/LHY* amplitude was relatively smaller and had a similar profile as those in model M2 (Supplementary Figs S2 and S6).

Because of several IFFL-2s working together in a complete network of model A, whether the pulse-like expression of *PRR9/7* is really caused by the IFFL-2 from *CCA1/LHY* to *PRR9/7* is unclear. Thus, we compared the *PRR9/7* expression profile in both the wild-type and mutant condition (Fig. 4a) and measured the full width half maximum (FWHM, normalized by the oscillation periods) of the expression peak for *PRR9/7* in both the wild-type and mutant condition (Fig. 4d, Method). For 83.51% of selected parameter sets, the normalized width of *PRR9/7* was smaller in the wild-type than mutant condition (Fig. 4e). These results indicate that the IFFL-2 from *CCA1/LHY* to *PRR9/7* is really generating pulse-like expression of *PRR9/7* in the complete network of model A.

Finally, we hypothesized that if the IFFL-2 is important, it cannot be replaced by *CCA1/LHY* auto-positive feedback loop, which can increase the *CCA1/LHY* amplitude and delay *PRR9/7* expression time but cannot induce pulse-like expression of *PRR9/7*. Therefore, we created another model, called Pos-CCA, which consisted of the *CCA1/LHY* auto-positive feedback loop (but without the IFFL-2), and compared it with models A (with IFFL-2) and M2 (without IFFL-2) (Fig. 1a and Fig. 4a). Our simulation results showed similar normalized hitting rates among these three models (Fig. 4f), but the performance on genetic perturbation tests (*cca1/lhy*, *prr9/7*, *prr5/toc1*, see methods for detailed information) in the new model were greatly reduced as compared with model A (Fig. 4g). Therefore, the IFFL-2 from *CCA1/LHY* to *PRR9/7* is important for replicating the correct dynamics of the core circadian oscillator.

### Evening complex completes the switching process

**Figure 5:**
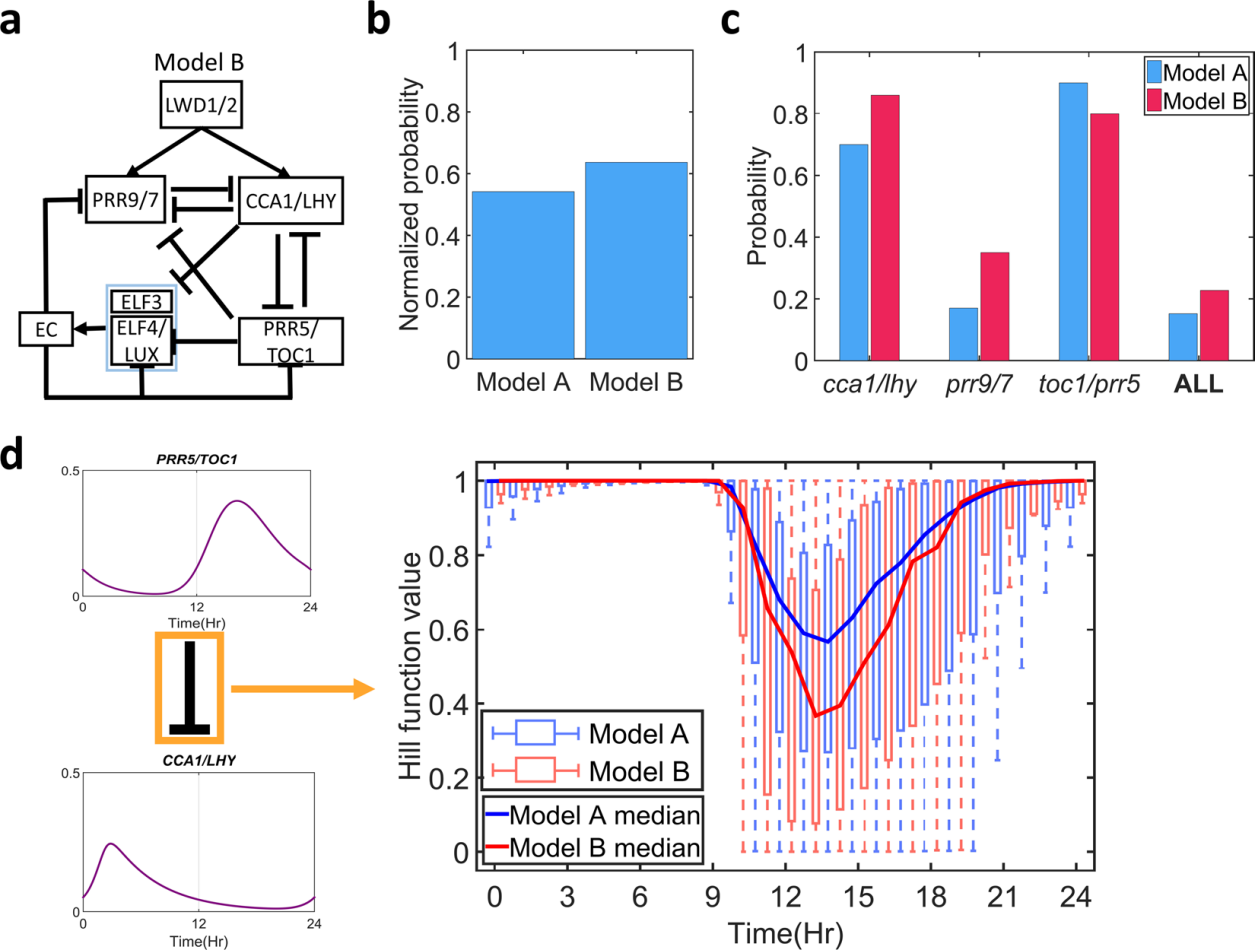
Adding *EC* improved the robustness and performance of the *Arabidopsis* clock. (**a**) Schematic representation of model B. (**b**) The averaged hitting rates of each parameter showing regular oscillation and correct *lwd1/2* mutant dynamics in model A and model B. (**c**) The probability that the obtained parameter sets showed correct period changes in the genetic perturbation test results (*cca1/lhy*, *prr9/7*, *prr5/toc1*) for each model. (**d**) Hill function values for *PRR5/TOC1* inhibition of *CCA1/LHY* for all selected parameter sets that showed a correct genetic perturbation in model A (blue) or model B (red), presented as box plots. The blue and red solid lines represented the median Hill function value for models A and B, respectively.

Model A does not contain any evening genes. The system moves back with the negative feedback loop in the nighttime. We further studied a nighttime component, the evening complex (*EC*), which was found important in clock systems^37,38^. Because *ELF4* and *LUX* genes are regulated similarly, following the previous mathematical models^47,48,51^, they were combined and represented as *ELF4/LUX* to reduce the model complexity. The discrepancy in many mathematical models in describing *EC*^47,48,51^ did not lead to significant differences in model dynamics (Supplementary Fig. S7). Thus, to accommodate all observed experimental results, in model B, the *EC* is represented by a complex of two genes, *ELF3* and *ELF4/LUX*, which is repressed by *CCA1/LHY* and *PRR5/TOC1* and which repressed *PRR9/7*, *PRR5/TOC1* and its own expression (Fig. 5a).

#### EC forms an additional repressilator that improved the robustness and performance of the Arabidopsis clock

We found a noticeable improvement when adding *EC* into the system. Our simulations showed that the hitting rates and performance on the genetic perturbation test for model B were increased more than 20% and 30%, respectively (Fig. 5b,c). Furthermore, the improvement in genetic perturbation test performance was mainly due to a better performance of the *prr9/7* mutant, with increase more than 60% (Fig. 5c). This addition of EC actually added another repressilator in the clock system (Fig. 5a). The weak *PRR5/TOC1* inhibition of *CCA1/LHY* in model A (higher threshold [κ] values, Supplementary Fig. S5B) was now released by the additional repressilator loop formed by *EC*, which allowed for stronger *PRR5/TOC1* inhibition of *CCA1/LHY* (Fig. 5d). Moreover, it led to a stronger double-negative feedback loop and stronger bistability. This stronger bistability accentuated the roles of *PRR9/7* (to break the bistability), shown by better genetic perturbation test performance.

#### EC is also able to break the bistability but does not work as a rapid switcher

**Figure 6:**
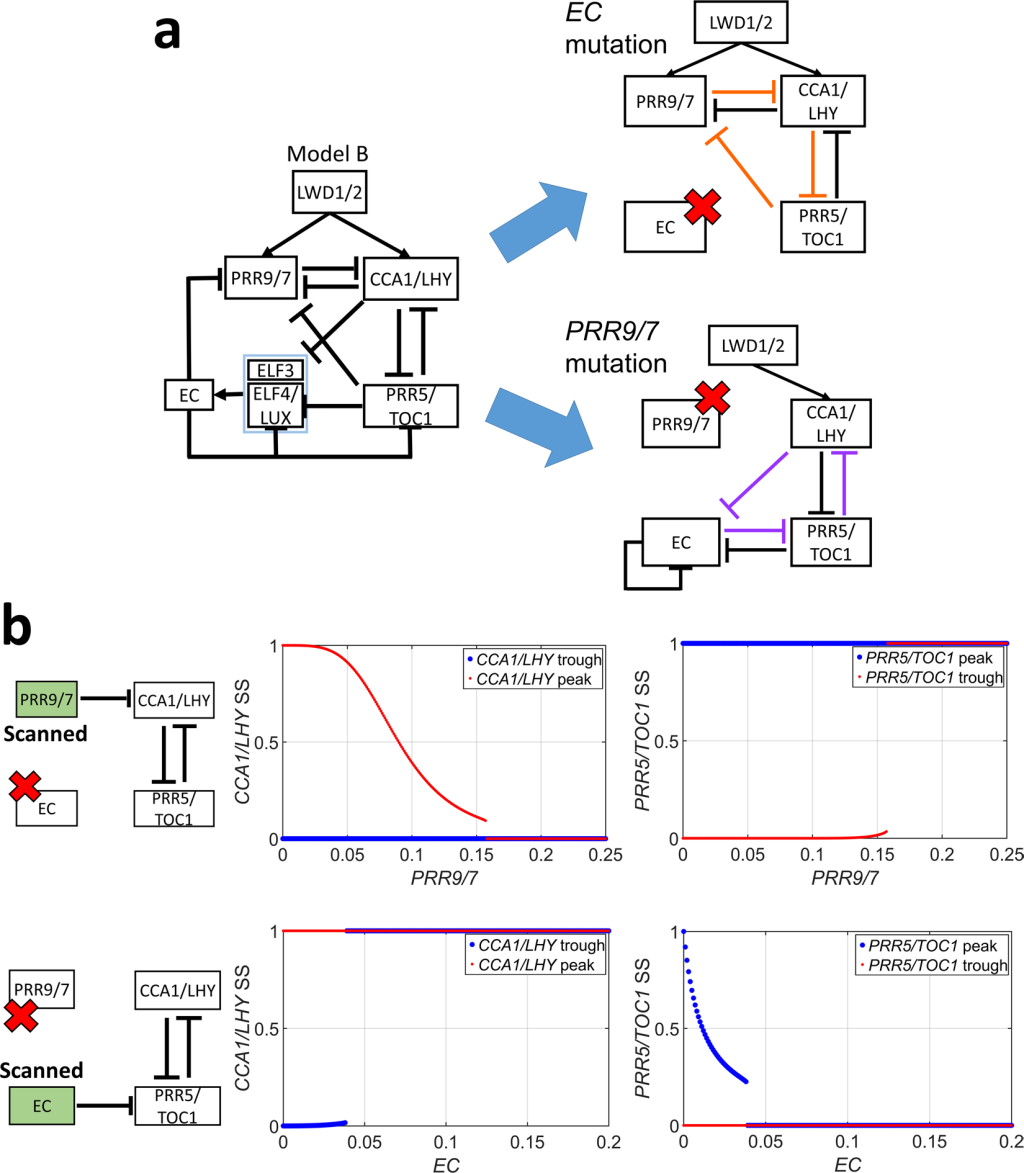
The symmetrical structure of model B. (**a**) Schematic representation of similar networks formed by mutating *EC* (upper panel) or *PRR7/9* (lower panel). (**b**) The dynamics of the tested partial network. Dots represent the steady-state value of *CCA1/LHY* level (middle panel) or *PRR5/TOC1* level (right panel) starting from a low level of *CCA1/LHY*/ high level of *PRR5/TOC1* (blue dots), or high level of *CCA1/LHY*/low level of *PRR5/TOC1* (red dots).

When *PRR9/7* was mutated, the network structure of model B was very similar to model A but in the opposite direction (Fig. 6a). This symmetry led us to hypothesize that *EC* might also break the bistability in the opposite direction. We performed a similar analysis, by mutating *PRR9/7* and *EC* simultaneously, then added one of them back as an input (Fig. 6b left). The steady state of *CCA1/LHY* (Fig. 6b middle) or *PRR5/TOC1* (Fig. 6b right) was gathered as a function of *PRR9/7* or *EC*, respectively (Method). EC indeed broke the bistability. The increase in *EC* gradually turned *PRR5/TOC1* off similar to *CCA1/LHY* being turned off when *PRR9/7* was varied (Fig. 6b). Moreover, this behavior was found for 63.44% of selected parameter sets obtained in model B (Supplementary Fig. S8), which indicated that it occurs due to the dynamics of the system instead of certain parameter values.

Next, we noted that *EC* was also under the influence of IFFL-2 as observed previously for *PRR9/7* (Fig. 6a). Hence, we conducted a similar analysis as was done previously in model A (Method). Unfortunately, although we still could observe pulse-like expression of *PRR9/7* in model B, we could not find pulse-like expression in *EC* (Supplementary Fig. S9). The only difference between these two genes is that *EC* was also controlled by an auto-negative feedback loop (NF), and *PRR9/7* was not. Therefore, we tested the effect of adding an auto-negative feedback loop to the ability of IFFL-2 for generating pulse-like expression by comparing two models consisting of only IFFL-2 (Supplementary Fig. S5A) or IFFL-2 + NF (Supplementary Fig. S10). The addition of an auto-negative feedback loop into IFFL-2 greatly reduced the ability of IFFL-2 in generating pulse-like expression (Supplementary Fig. S10). These results might explain at least in part why *EC* failed to generate pulse-like expression in model B.

#### EC works as the nighttime switcher in the clock system

**Figure 7:**
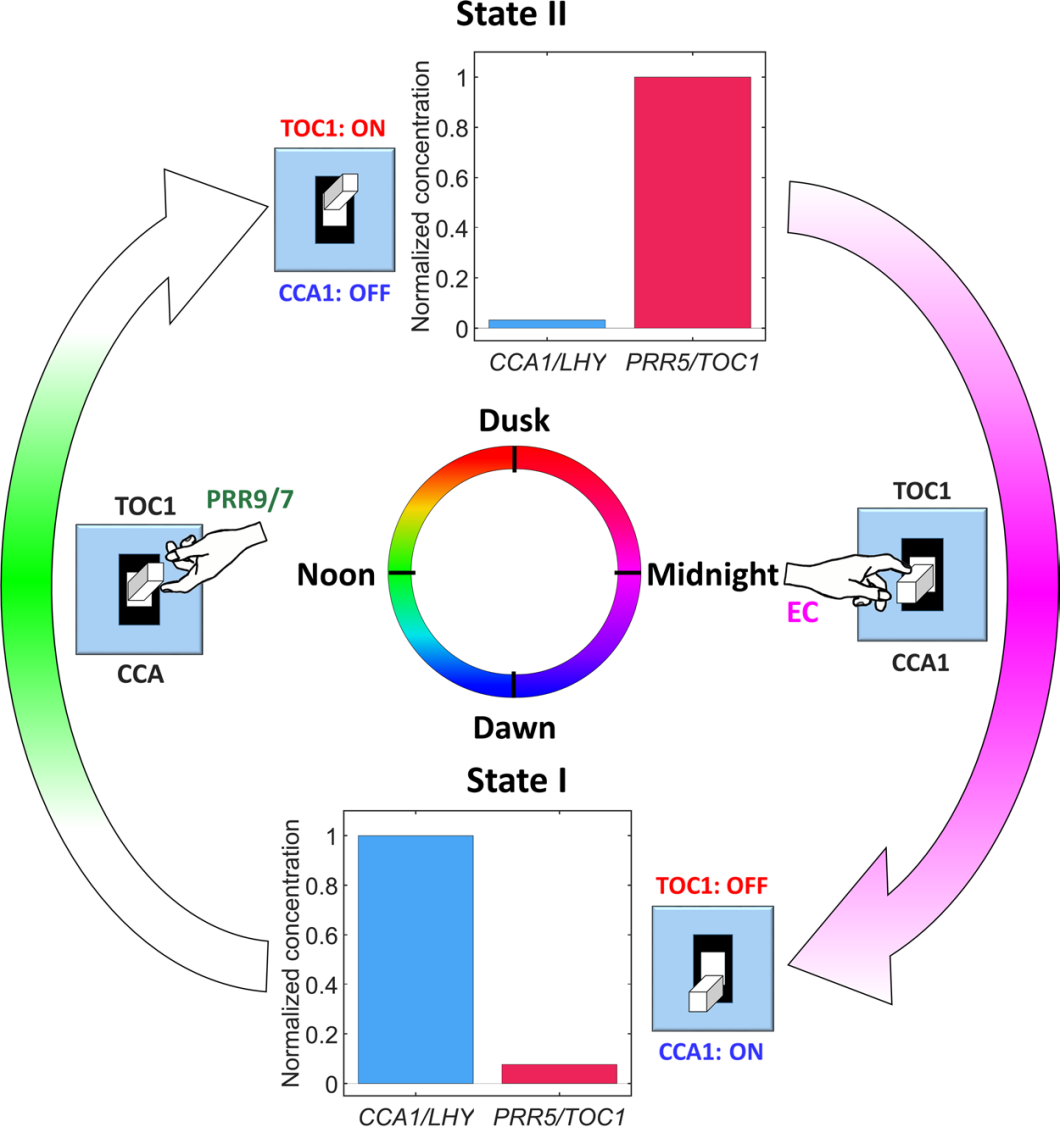
Schematic representation of the dynamics of the Arabidopsis clock. *CCA1/LHY*, *PRR9/7*, *PRR5/TOC1*, and *EC* genes are indicated by blue, green, red, and magenta, respectively. Arrow intensity represents the *PRR9/7* or *EC* expression level throughout the day or night, respectively.

Therefore, *EC* completes the switching process by turning *PRR5/TOC1* off (and breaks the bistability) in the nighttime. To illustrate this idea, we divided the system into two different states during the 24-hr cycle and highlighted the system dynamics at that particular state (Fig. 7). At dawn (State I), the system stops at one of the bistable states when *CCA1/LHY* expression is at the peak level and the expression of *PRR5/TOC1* is low. *PRR9/7* expression peaks near noon, reduces the concentration of *CCA1/LHY*, which in turn, promotes the accumulation of *PRR5/TOC1*. Next, near dusk, the system reaches the other bistable state when *PRR5/TOC1* reaches its peak level while *CCA1/LHY* is kept at a low concentration (State II). Because of the low *CCA1/LHY* level, *EC* accumulates fast and peaks near midnight. Consequently, this *EC* accumulation reduces *PRR5/TOC1* concentration, which in turn, promotes *CCA1/LHY* accumulation. Thus, *CCA1/LHY* once again peaks in level near dawn (state I), which completes the daily cycle of the clock system.

#### Bistable and switching dynamics are general, not limited to current network structures

To rule out the possible limitations in using this simplified model, we performed a similar analysis with more comprehensive and established models and sought a similar behavior described previously (Method). We chose P2012 and F2014 models, which include many known clock genes, can replicate the multiple genetic perturbations observed in the experimental results, and contain more details in describing *PRRs* and *EC* genes in the clock system^47,48^. Furthermore, P2012 has been extended to simulate and interpret the input^64^ and output^65^ pathways. Under *PRR9* titration, the *CCA1* and *TOC1* genes also generated a hysteretic bistability in both models (Supplementary Fig. S11A,B). However, under *EC* titration, only F2014 generated hysteresis (Supplementary Fig. S11B). In P2012, when we added a constant input of *EC* protein under the *EC* mutant condition, the system oscillated mildly, which prevented us from obtaining the steady state concentration of *CCA1/LHY* and *TOC1* genes (Supplementary Information).

Next, we also tested whether *PRR9* and *PRR7* expression exhibits pulse-like behavior in both systems by performing a similar analysis described previously (Method). Unfortunately, in P2012, the *PRR9* and *PRR7* genes were still under direct activation of *CCA1/LHY*. Hence, we could only test the pulse-like behavior of *PRR9* and *PRR7* expression in F2014. Only *PRR7* but not *PRR9* showed pulse-like expression due to IFFL-2 (Supplementary Fig. S12). In F2014, *PRR9* was weakly repressed by *CCA1* and *LHY*, as stated in the original paper^48^, and thus less influence of IFFL-2 is not surprising. Therefore, these results suggest that the insights we derived from simplified models can also be seen in more complete and detailed models in the literature.

## Discussion

### The IFFL in the clock system

Unlike the coupled positive and negative feedback loops, IFFLs are rarely discussed as a core network motif in the clock. These IFFLs are abundant in the *Arabidopsis* clock (Figs 3 and 5a). For instance, model A can actually be seen as a combination of two interlinked IFFL-2s, which start from *CCA1/LHY* to *PRR9/7* and *PRR5/TOC1* to *CCA1/LHY* (Fig. 3). This interlinked IFFL is sufficient to create another network motif such as a repressilator and positive feedback loop. In accordance with our observations, previous study showed that IFFLs are actually the most frequently occurring potential motifs in the mammalian clock (with 358 possible occurrences)^39^. Thus, studying the roles of these IFFLs inside the clock system is becoming unavoidable.

In this study, we demonstrated that IFFL-2 is able to turn *PRR9/7* into a rapid switcher, which is important for replicating the correct dynamics of the clock system. *PRR9* and *PRR7* genes have been shown to play many important roles in gating environmental signals, such as light, temperature, or sugar, into the clock system^66–68^. Having a pulse-like expression on these genes might be beneficial for the clock, since it can enable them to response rapidly in facing sudden changes of environmental signal. Moreover, this pulse-like expression also narrows down the expression time of *PRR9* and *PRR7* genes, so they express at a precise time window, which might be important for many downstream genes. Previously, LHY expression was also shown to be under the regulation of an IFFL, which is formed by *GI*_*N*_, *GI*_*C*_, and *LHY* and plays important roles in ensuring the robustness of *LHY* amplitude and phase^69^.

Temperature compensation is an important property of circadian rhythms^12^. Recent computational study showed that IFFLs have inherent features that promote temperature robustness. Furthermore, an additional auto-negative feedback loop on the target gene of the IFFL network enhances the temperature robustness properties^45^. In this study, we found that unlike *PRR9/7*, *EC* does not generate any pulse-like expression because it has an additional auto-negative feedback loop (as discussed previously). In addition, several studies have reported that *EC* might work as an input gateway of the temperature signal to the clock^37,70^. Thus, this temperature robustness could be studied more closely in the future.

Moreover, IFFL has been studied and reported to provide “fold-change” detection under specific parameter settings^43,44^. Another interesting property of the IFFL is its band-pass filter, which allows a gene to respond only to a specific periodic signal^71,72^. Recently, the IFFL has also been found to double the frequency of cyanobacteria circadian rhythms^73^. How these special properties of the IFFL work in the plant’s clock is unclear. Our current report would certainly help future development in understanding the unique character of the plant’s clock.

### Coupled positive and negative feedback loops are commonly seen in biological oscillators

In the present work, model simulations show that a negative feedback coupled with a positive feedback loop can generate a toggle switching oscillation in the *Arabidopsis* clock system. This coupled feedback loop system is also commonly seen in the clock of many model organisms, such as mouse, fruit fly, and fungi. For example, in the mammalian clock, the core oscillator is driven by *BLMA1/CLOCK* activation to *PER/CRY* and the subsequent negative feedback of *PER/CRY* into its own activation. This core oscillator is supported by an ancillary loop involving *Rors* and *RevErbs*, which forms positive and negative feedback loops, respectively^3,4,39,74^. Similarly, both positive and negative feedbacks are found in the *Neurospora* circadian clock. In *Neurospora*, the core oscillator is activated by *WCC* activation to the *FRQ/FRH* protein complex (*FFC*), which in turn inhibits this activation and forms a negative feedback loop. However, unlike the mammalian clock, *FFC* also directly activates the expression of *WC-1*, thereby creating a positive feedback loop in the clock system^75–78^.

In contrast, the cyanobacteria clock is one exception whereby a positive feedback loop is not yet reported as an integral part of the core oscillator. In *Synechococcus elongates*, the core oscillator is driven by the auto-kinase and auto-phosphatase activity of *KaiC* by *KaiA* and *KaiB*, respectively. A solution with these purified proteins is sufficient to generate a robust 24-hr oscillation that is temperature-compensated^79^. However, phosphorylated *KaiC* actually activates *RpaA*, an activator of *KaiB* and *KaiC*, which forms negative and positive feedback loops, respectively, to *KaiC*^1^, but Kai proteins being able to robustly oscillate in the absence of *RpaA* prohibited us from speculating even further.

Therefore, the coupled positive and negative feedback loops in the clock oscillators are seen in the clock of many organisms. Whether this coupled feedback loop can also generate a hysteretic switch or toggle switching for other clock systems remains for investigation.

### The advantage of a hysteretic switch bistability in the system

Many studies have shown that bistability is present in both oscillating and non-oscillating systems^14–19^. A good example of an oscillating system with bistability is the cell cycle oscillator of *Xenopus laevis*. In this system, the response of *CDC2* to cyclin is hysteretic, forming two stable states of *CDC2* activity depending on the initial condition of *CDC2*^14^. As well, a bistable positive feedback system coupled with a negative feedback loop could yield self-sustaining, spike-like oscillations of a relaxation oscillator^14^. Moreover, a computational study showed that with a strong positive feedback, an oscillator is widely tunable in frequency and with nearly fixed amplitude. This tunability is important in many biological rhythms such as heartbeats and the cell cycle^19^. However, we did not observe a similar tunability in the frequency of the *Arabidopsis* clock.

In another study, hysteresis has been shown to generate robust oscillation with a large correlation time and a small variation in period length. This robust oscillation could not be achieved in the time-delay, non-hysteretic, oscillator^18^. Therefore, the presence of hysteresis might help the *Arabidopsis* clock oscillate robustly despite variations in the cell or in the environment.

In a non-oscillating system, bistability has been observed in many systems such as the lactose uptake system in *E. coli*^15^, galactose-uptake system in yeast^16^, and cell migration system in breast cancer cells^17^. In the lactose and galactose-uptake systems, this hysteresis can filter out noises present in the input signal or provide a memory, such that the system will only turn on/off when the signals truly persist. Consequently, the hysteresis lowers the metabolic cost of reinitiating the synthesis of the uptake machinery after a transient fluctuation, which enhances the fitness of the cells^15,16^. Therefore, the hysteretic switch in the clock may filter out the noises in the environmental signal, such that the clock can only be reset by true persisting signals.

### The addition of several redundant feedback loops might be important for the robustness of the *Arabidopsis* clock

In recent study, *Marchantia polymorpha* has been shown to only have one single homolog of each important clock genes, such as *CCA1*, *PRR*, *TOC1*, *ELF3*, *ELF4* and *LUX*, which is highly redundant in *Arabidopsis*^80^. In relation to our study, we merged functionally similar genes in the *Arabidopsis* clock system and represented it as a single gene. The representation of model B is actually quite similar to the number of genes found in *M. polymorpha* clock. Under daily light and dark (LD) cycles, the *M. polymorpha* clock was able to oscillate robustly. However, in contrast with robust oscillation in the *Arabidopsis* clock, only week rhythms were detected for *M. polymorpha* clock under constant light (LL) or dark (DD) condition^80^. Unfortunately, the interactions of each gene in the *M.polymorpha* clock are still unknown, which prevent us to do a more elaborate comparison in the behavior of *Arabidopsis* and *M.polymorpha* clock system. On the other hand, these results can provoke one’s curiosity whether additional redundant feedback loops might be needed for *Arabidopsis* clock to be more resilient in the noisy environmental condition. Thus, further studies on the clock of earlier land plants will be valuable in giving us better understanding on how the clock system might work.

### Simplified model is useful for insight into a complex system

Our simplified models could represent the essential dynamics of the *Arabidopsis* clock. As summarized in Fig. 7, the clock system is like a regular toggle switch formed by the bistability in the feedback loop of *CCA1/LHY* and *PRR5/TOC1*. Furthermore, these two stable states, labelled *CCA1 ON* and *TOC1 ON*, switch alternately in the plant with periodicity of approximately 24 hr. However, switching from one state to another requires a driver (pictured as a moving hand) that breaks the stability and moves the system forward. In the *Arabidopsis* clock, *PRR9/7* and *EC* serve as the switchers, which break the bistability and switch the states in the daytime and nighttime, respectively. Moreover, this illustration helps in understanding certain mutant behaviors observed in the experimental results as we discussed above.

Thus, our work provides an example of how a simplified model can be used to understand the behavior of complex systems. With a reduced number of equations and parameters, we can easily dissect and manipulate the core regulatory mechanism of the clock. Furthermore, a more general random search and steady-state analyses can be applied easily to the simple model, which is more difficult to achieve in the more detailed models.

However, with a simplified model, we obtain more qualitative rather than quantitative conclusions. Simplified models can only replicate the relative expression of the clock genes. Using our model, we could not replicate the precise phase and trajectory of all the clock genes as shown in the experimental results (Supplementary Fig. S13). This results is similar with the previous observation seen in the C2016 model^51^. However, this limitation can be solved easily by adding additional components or regulations, as shown in the P2012 and F2014 models^47,48^. Therefore, depending on the purpose of the study, both simplified and detailed models can be used to gain different levels of understanding the complex system.

## Material and Methods

### Model representation

All models are described by a set of ordinary differential equations (ODEs) for the simulation under continuous light (see Supplementary Information for more details). In general, each gene was represented as:

1 $$\frac{dX}{dt}=\beta .\,Hill-\gamma X$$

where x represents the protein concentration, which can be *CCA1/LHY*, *PRR9/7*, *PRR5/TOC1*, *ELF4/LUX*, or *ELF3* depending on the model, β represents the total production rate, and γ is the degradation rate. Hill represents the Hill function, which describes the effects of upstream regulation as

2 $$hil{l}_{act}=\frac{{[Activators]}^{n}}{{\kappa }^{n}+{[Activators]}^{n}}$$

for the activating process and

3 $$hil{l}_{rep}=\frac{{k}^{n}}{{\kappa }^{n}+{[Repressor]}^{n}}$$

for the repression process. Following previous studies, we also use an ‘AND’ gate to describe a combination of two or more source of regulations, where the two Hill functions are multiplied^47,48,51^.

### Searching, propagation, and selection process

All independent parameters in each model were obtained by random searches, propagated, and screened for regular oscillation. The search was performed at a logarithmic scale across three orders of magnitude, for γ’s and κ’s, and a linear scale for *α*’s (Supplementary Table S2). The criteria we used are as follows:

1. The trajectory must oscillate regularly, defined by examining the period and amplitude change in each cycle. We calculated the relative difference in period and amplitude change for each cycle, defined as |(*x*_1_ − *x*_*2*_)|/min(*x*_1_, *x*_2_), where *x*_1_ and *x*_2_ are the period or amplitude calculated from two consecutive cycles. An acceptable regular oscillation was defined as that with less than 5% relative change for more than 10 cycles.
2. In the *lwd1/2* mutant, the oscillation must have reduced amplitude (>50%) and shorter period (<21 hr), as reported previously^20^.

For all searching, we used similar initial conditions, which is 10% of maximum possible steady-state concentration. Finally, all simulations were performed by using both MATLAB (The MathWorks Inc., Natick, MA) and Octave 4.0.0^81^.

### Hitting rates calculation

In comparing the hitting probability, an “averaged” probability of hitting for each parameter was calculated as,

4 $$P=\sqrt[n]{\frac{\#\,of\,obtained\,parameter\,sets}{\#\,of\,searched\,parameter\,sets}}$$

where *P* represents the averaged probability per parameter and *n* is the number of independent parameters^82^. A model was called more robust if it has higher *P*.

### Genetic perturbation test

To simulate mutants, it would be ideal to simply turn off the expression of the corresponding gene(s). It is typical to observe shorter- or longer-day mutants, which we aimed to reproduce with our models. However, in the simplified model studied for the core oscillator, the system sometimes no longer oscillated when the core negative feedback was disrupted. Considering potential functionally similar genes and pathways in the organism, we performed the genetic perturbation tests by scaling the gene production rates (*β*) down by a fraction, systematically scanned from 1 to 0, while keeping the same degradation rates (*γ*). For each parameter set, we monitored the period changes at the scanned perturbation level. A parameter set was labeled as “correct” if any of the genetic perturbation tests produced a shorter period by more than 3 hr under *cca1/lhy* perturbation^26^, a longer period by more than 1 hr under *prr9/7* perturbation^32^, and a shorter period by more than 3 hr under *prr5/toc1* perturbation^62,83^.

### CCA1/LHY overexpression tests

Similar to genetic perturbation tests, the consecutive overexpression was done increasing the total production rate (*β*) of *CCA1/LHY* while keeping the other parameter values the same. Here we systematically scanned for the fraction of production rate (from 1 to 2, with 0.01 increments), where “1” represents the wild-type condition. After that, the *PRR9/7* amplitude was calculated and compared to the wild-type condition. Parameter sets with higher *PRR9/7* amplitude were used for each model.

The transient overexpression was performed by changing the initial condition of *CCA1/LHY* while keeping all other parameters the same. Because the level of transient overexpression determined the phased shift that occurred in *PRR9/7* expression (Supplementary Information), for data representation in Fig. 1f, we randomly chose a parameter set and fine-tuned its overexpression level. Here, the initial value of *CCA1/LHY* was increased 700-, 500-, and 2-fold the maximum steady state value of model M1, M2, and M3 (model A), respectively. After that, to show the overall behavior (Supplementary Fig. S4), we fixed the overexpression level of *CCA1/LHY* (5-fold the maximum steady state value) and then applied it to all parameter sets in each model. Next, we compared the *PRR9/7* expression at 2 hr after the induction and the first *PRR9/7* amplitude after the induction with the wild type. Parameter sets with lower expression at 2 hr after induction and higher amplitude of *PRR9/7* after induction were used.

### Hysteresis analysis

The hysteresis analysis was performed by involved mutating *PRR9/7* and/or *EC* genes, then adding one of them back as a constant input at various concentrations. Here, the concentration of the gene was systematically scanned from 0 to 1, with 0.001 increments, where “0” represents the total absence and “1” the maximum steady state value of the input gene for each parameter set. After that, the simulation was propagated from two different initial conditions and the steady state value of *CCA1/LHY* (and *PRR5/TOC1* for model B) was measured at the end of the simulation. The first initial condition was started from a high initial concentration of *CCA1/LHY* (at the peak value of the wild-type oscillation), and the second initial condition was started from a low initial concentration of *CCA1/LHY* (at the trough value). If the *CCA1/LHY* and *PRR5/TOC1* had two different concentrations at a certain concentration of *PRR9/7* or *EC* (for model B), then it was counted as a parameter set that shows bistability. A model was called to have stronger bistability if they have wider bistability region.

### Incoherent feed-forward loop analysis

In this study, the ability of IFFL to generate a pulse-like expression was tested in both the partial and complete network.

For partial network analysis, *CCA1/LHY* (Fig. 3a) or *PRR5/TOC1* (Fig. 3b) was mutated and, as a replacement, an input function represented the mutated gene was added. Here, we started the input function from an initially high concentration (maximum concentration) before gradually turning it off at t_off_, with the rate of $\,{e}^{-\gamma {t}^{\text{'}}}$. Here, γ represents the degradation rates of the mutated gene with given parameter sets and t’ represents the time after the mutated gene was turned off (t′ ≡ t − t_off_). All local maxima were then identified and screened based on their prominence level. Peak prominence was defined as the height of the peak compared to the highest nearby local minima. Moreover, to rule out unwanted numerical error, we added a stringent criterion for defining parameter sets that showed pulsing behavior such that only those showing a prominence to peak ratio >0.5 were used. This relative threshold was easier to handle than an absolute threshold considering the wide range of parameter values that we obtained through random sampling.

Next, the complete network analysis was performed in two different ways: the delayed time analysis, and the analysis for the pulse. For both analyses, each parameter set was initially propagated until it reached stable oscillation and then the time when *PRR5/TOC1* reached its peak value was chosen (Fig. 4b when t < 0). We used the concentration of all genes at this time window (*PRR5/TOC1* peaking time) as an initial condition for the second propagation (Fig. 4b at t = 0). Furthermore, for the delayed time analysis, we measured the time when the *PRR9/7* concentration reached half-maximum for the first time in both the wild type (WT, denoted t_1_) and mutant condition (denoted t_2_). The delayed time (τ) was defined as the time difference between t_2_ and t_1_ (Fig. 4b). However, for the pulse analysis, we measured the full width at half maximum (FWHM) of the first peak in both the WT and mutant condition. In this study, a system with shorter expression time is considered as more pulse-like than that with longer expression. Since the mutation affected the oscillation period, each width was further normalized by using their periods (T) (Fig. 4d). Using this approach, a lower normalized width value indicates more pulse-like behavior in the target gene.

### Comparison with other models

We also performed the analyses for hysteresis and pulse-like behavior for previously published models^47,48^. For the hysteresis analysis, we first performed a simulation under the *prr9;prr7* double-mutant condition in the entrainment (LD) and then released to a constant light condition (LL). We then “froze” the simulation at two different time points, one at a high concentration of *CCA1/LHY* (peaking time) and the other at a low concentration of *CCA1/LHY* (trough time), and recorded all gene concentrations at that particular time window. Next, we mutated the *LUX* gene to break the clock rhythmicity and added a constant input of *PRR9* or *EC* protein back into the system. Here we systematically scanned for the concentration of the gene (from 0 to peak value, with 0.001 increments), where “0” represents the totally absent and peak value represents the high expression of the *PRR9* or *EC* protein. Finally, the simulation was propagated from these two different initial conditions (with various levels of *PRR9* or *EC* protein), and at the end of simulation, the *CCA1/LHY* and *TOC1* steady-state value was measured. All parameter values followed the original parameter sets provided by the authors in their manuscripts (for Fogelmark’s model parameter set No. 5)^47,48^.

For the pulse-like analysis of F2014, we again calculated the FWHM of the first peak in both the WT and mutant conditions. In the mutant condition, we mutated the *CCA1* and *LHY* inhibition to *PRR9* (Supplementary Fig. S12A) or to *PRR7* (Supplementary Fig. S12B). All parameter values followed the original parameter sets provided by the author in their manuscript (parameter set No. 5)^48^.

## Electronic supplementary material

Supplementary Information

Supplementary dataset

## Data Availability

All data generated or analyzed during this study are included in this published article (and its Supplementary Information files).
