# Supplementary material for: An incoherent feed-forward loop switches the Arabidopsis clock rapidly between two hysteretic states
Source: Sci Rep. 2018 Sep 17;8:13944. doi: 10.1038/s41598-018-32030-z (PMC6141573; doi:10.1038/s41598-018-32030-z)
Supplement: Supplementary file 1 — Supplementary Information [file 41598_2018_32030_MOESM1_ESM.pdf]

Supplementary Material for:

An incoherent feed-forward loop switches the Arabidopsis clock rapidly between two hysteretic states

Ignasius Joanito[1,2], Jhih-Wei Chu[2,4], Shu-Hsing Wu[3,5], Chao-Ping Hsu[1,5*]

[1]Institute of Chemistry, Academia Sinica, Taipei, 11529, Taiwan
[2]Bioinformatics Program, Taiwan International Graduate Program, Academia Sinica, Taipei, 115, Taiwan and Institute of Bioinformatics and System Biology, National Chiao Tung University, Hsinchu, 300, Taiwan
[3]Institute of Plant and Microbial Biology, Academia Sinica, Taipei, 11529, Taiwan
[4]Department of Biological Science and Technology, National Chiao Tung University, Hsinchu, 300, Taiwan
[5]Genome and Systems Biology Degree Program, National Taiwan University, Taipei, 106, Taiwan
*Correspondence to: Chao-Ping Hsu, Email: cherri@sinica.edu.tw, Fax: 886-2-2783-1237

Table of Contents

1. Model Simplification 2
2. Supplementary methods 3
   a. Model M1 3
   b. Model M2 5
   c. Model M3 (Model A) 5
   d. Model Pos-CCA 5
   e. Model M4 (Model B) 6
3. Supplementary results 6
   a. Adding direct activation of *LWD1/2* to *PRR5/TOC1* reduced the performance of model M1 6
   b. The direct inhibition of *CCA1/LHY* to *PRR9/7* is important for correct *prr9/7* genetic perturbation test result 7
   c. Under *CCA1/LHY* overexpression, the expression of *PRR9/7* was elevated in all models 8
   d. Only model model A shows an immediate reduction of *PRR9/7* expression under transient overexpression of *CCA1/LHY* 9
   e. *PRR5/TOC1* generates pulse-like expression of *CCA1/LHY* gene and not *PRR9/7* 10
   f. The IFFL-2 from *CCA1/LHY* to *PRR9/7* helps increase *CCA1/LHY* expression level 11
   g. Different *EC* representation has little impact on model performance and dynamics 12
   h. The insights we obtained from model A can also be seen in model B 13
   i. Our findings are general and are not limited to certain model structures 15
   j. The comparison of real experimental data with the simulated result found in model A 17
4. Supplementary Tables 18
   a. Table S1. Probability of finding proper parameter sets of all models 18
   b. Table S2. Search ranges for parameters 18
5. Supplementary References 19

1. Model Simplification

Considerations for merging genes and simplifying models.

The Arabidopsis circadian clock is a complex system that involves many genes. More than 20 clock or clock-associate genes have been identified for *Arabidopsis thaliana* [1]. However, some have a similar function in the clock system. Therefore, in this study, we combined functionally similar genes in the clock systems to reduce model complexity. Moreover, to reduce the model complexity even further, we focused our simulations on only the constant light condition (LL) because we aimed to derive insights for the underlying mechanism of the intrinsic clock.

CIRCADIAN CLOCK ASSOCIATED1 (CCA1) and *LONG ELONGATED HYPOCOTYL (LHY)* are MYB-like transcriptional factors with similar expression profile and partially, if not all, redundant roles in the clock systems [2]. These genes are repressed by *PSEUDO-RESPONSE REGULATOR* (PRR) family genes, *PRR9*, *PRR7*, *PRR5*, and *PRR1/TOC1* [3-5], and in return, they repress many evening clock genes such as *PRR5*, *TOC1*, *EARLY FLOWERING 3* (*ELF3*), *ELF4*, and *LUX ARRHYTHMO* (*LUX*) [6-9]. Furthermore, *CCA1* and *LHY* could also bind to the *PRR9* and *PRR7* promoter region and have a positive effect on their expression [10]. However, whether this positive effect is from direct or indirect activation is unclear [11]. Therefore, we represented *CCA1* and *LHY* genes as a single component, *CCA1/LHY*, following previous mathematical models [12-15], which also can reduce model complexity.

Next, the PRR family genes have been reported to play important roles in the clock systems and were retained in our model. These family proteins consist of five genes, *PRR1/TOC1*, *PRR3*, *PRR7*, *PRR5*, and *PRR9*. These genes are expressed in sequential order and form a "*circadian waves of APRR1/TOC1 quintet*" [16]. In earlier study, *TOC1* and *CCA1/LHY* were widely believed to be the core oscillator that drives the oscillation in the clock system [6,16,17]. However, in the following years, *TOC1* was found to work as an inhibitor instead of an activator of *CCA1/LHY* [4,5]. This mutual inhibition of *CCA1/LHY* and *TOC1* formed a double-negative or a positive feedback, in the clock system. In many organisms, positive and negative feedback loops play important roles in driving the oscillation process [18]. Moreover, a triple mutation of *prr9;prr7;prr5* genes led to an arrhythmic behavior, which further strengthened the importance of these family proteins. However, *PRR3* was excluded from our model because it is only expressed in vascular cells [19].

Many studies have shown that *PRR5* and *TOC1* are similar in transcriptional and post-translational regulation. At the transcriptional level, both are activated by a MYB-like transcriptional factor, *REVEILLE8 (RVE8)*, and are inhibited by *CCA1* and *LHY* [6,20,21]. Another experiment shown that in *lwd1;lwd2* mutant plants, both *PRR5* and *TOC1* are strongly upregulated, whereas *PRR9* and *PRR7* are strongly downregulated, which again implies that these two genes might be under similar transcriptional control [22]. At the post-translational level, *PRR5* and *TOC1* proteins are tagged by an F-box protein, *ZEITLUPE (ZTL)*, and then degraded through 26S proteasomes pathways in the night [23,24]. *PRR5* and *TOC1* also form a dimer

that enhances the TOC1 nuclear accumulation, so their activity might tightly linked [25]. Furthermore, the loss-of-function mutant of these two genes showed a similar behavior, which led to a shorter period rhythm of the clock genes [6,26,27]. Therefore, these two genes were combined and represented as *TOC1/PRR5*.

In addition, both *PRR9* and *PRR7* have similar regulation. Earlier study showed that *CCA1*, *LHY*, and *LWD1* can bind to the *PRR9* and *PRR7* promoter region and have a positive effect on it [10,28]. In turn, *PRR9* and *PRR7* bind to the *CCA1* and *LHY* promoters and inhibit their expression [3]. At the post-translation level, both *PRR9* and *PRR7* proteins are degraded faster in the night, although the exact mechanism is still unknown [29,30]. Moreover, the loss-of-function mutant of these two genes also showed a similar, longer-period phenotype [10,31]. Thus, in this study, these two genes were combined and represented as *PRR9/7*.

The degradation of *PRR5* and *TOC1* is mediated by two other clock proteins, *GIGANTEA* (*GI*) and *ZTL* [23,24,32]. *ZTL* is an F-box protein that degrades *PRR5* and *TOC1* proteins via 26S proteasomes in the night. Furthermore, one previous work showed that GI is essential to establish and sustain the oscillation of *ZTL* protein by a direct protein–protein interaction, which stabilizes *ZTL* in vivo [32]. Since our simulations focused on constant light conditions, we excluded these two genes from our models for simplicity. Recent study showed that the inclusion of these two genes only slightly improved the description of the phase of *PRR5/TOC1* with no major qualitative changes. Moreover, the cost of adding them outweighed the benefits of the more accurate result [33]. Therefore, with similar reasoning, we also excluded two other clock genes, *CONSTITUTIVE PHOTOMORPHOGENIC 1* (*COP1*), and *RVE8* and its co-activator *NIGHT LIGHT INDUCIBLE AND CLOCK-REGULATED* (*LNK1/2*) [20,21,34,35].

Therefore, our first network structure, model M1, consisted of four groups: *CCA1/LHY*, *PRR9/7*, *PRR5/TOC1,* and *LWD1/2* (Fig. S1a left). In the early days, it was widely believed that *CCA1* and *LHY* directly activated the expression of *PRR9* and *PRR7*, and many mathematical models described this as an activator of *PRR9* and *PRR7* genes [14,15,36]. With this representation, the P2012 model was successfully simulated and interpreted the input [37] and output [38] pathways. Furthermore, model M1 has been used to show that *LWD1/2*, together with TCP proteins, are activators of *CCA1*. This model successfully represented the behavior of the clock system and could be easily integrated into a more comprehensive/detailed system [39]. Therefore, at first, we followed this idea and described *CCA1/LHY* as an activator of *PRR9/7* in our initial model (model M1). Later on, we changed the model structure to accommodate the new findings as described in the main text (model M2 and model A). Finally, we also incorporated the evening complex (*EC*) and tested its importance as described in the main text (model B).

2. Supplementary methods

a. Model M1

At the beginning, model M1 was composed of a set of three ordinary differential equations (ODEs) [equations (1) to (3)].

$$\frac{d[cca]}{dt} = \left(\alpha_{cca1}.\beta_{cca} + \alpha_{cca2}.\beta_{cca}.Hill_{act\_lwd}\right).Hill_{rep\_prr}\ .Hill_{rep\_toc} + \left(\alpha_{cca3}.\beta_{cca}\right).L.cP\ - \left(\mu_{cca1}.\gamma_{cca} + \mu_{cca2}.\gamma_{cca}.L\right).[cca], \quad (1)$$

$$\frac{d[prr]}{dt} = \left(\alpha_{prr1}.\beta_{prr} + \alpha_{prr2}.\beta_{prr}.Hill_{act\_lwd}\right).Hill_{act_{prr}} + \left(\alpha_{prr3}.\beta_{prr}\right).L.cP - \left(\mu_{prr1}.\gamma_{prr} + \mu_{prr2}.\gamma_{prr}.D\right).[prr], \quad (2)$$

$$\frac{d[toc]}{dt} = \beta_{toc}.Hill_{rep\_cca} - \gamma_{toc}.[toc]. \quad (3)$$

Here, [*cca*], [*prr*], and [*toc*] denote dimensionless concentrations of *CCA1/LHY*, *PRR9/7*, and *PRR5/TOC1*, respectively. The Nondimensionalization process was done by choosing a constant value for each component, denoted as X0. The X0 was defined as the ratio of total production rates / total degradation rates. In equations (1) to (3), β_x denotes the total production rate for gene *X*, and γ_x is for the total degradation rates. α's and μ's are dimensionless fractions of total rates from different regulation sources. For example, μ_{cca1} and μ_{cca2} are the fraction for the basal degradation and the additional degradation in the light condition for *CCA1/LHY*. *L* represents the light function (L = 1 when light is present and L = 0 otherwise) and *D* represents darkness ($D = 1 - L$). *CCA1/LHY* and *PRR9/7* were reported to accumulate rapidly in response to light [40,41]. Here, we followed previous work [13-15] and modeled this acute light response by using a light-sensitive activator protein *cP*. The expression of this hypothetical protein accumulates in the dark and degrades in the light. $Hill_{act}$ and $Hill_{rep}$ are the Hill input functions for an activator and a repressor, respectively:

$$Hill_{rep} = \frac{\kappa^n}{\kappa^n + [repressor]^n}, \quad (4)$$

$$Hill_{act} = \frac{[activator]^n}{\kappa^n + [activator]^n}. \quad (5)$$

As mentioned previously, "the Hill input function is a monotonic, S-shaped function, which is used to described the effect of the transcription factor on the transcription rate of its target gene" [42]. Here, κ represents the activation coefficient of activator or repressor and related to the chemical affinity between transcriptional factor gene and its site on the promoter region [42]. The n represent the Hill coefficient that governs the steepness of the input function, which related to ultrasensitivity process in the cell. There are many mechanisms leading to ultrasensitivity such as multisite phosphorylation, stoichiometric inhibitor, cooperativity, reciprocal regulation, and substrate competition [43]. Previously, several clock proteins have been shown to form a dimer [25,44,45]. Because of this reason, many mathematical models use n = 2, which is correspondence with this dimerization process [14,15,46]. However, in this study, we allowed n to be bigger than 2 to accommodate other possible regulation in the plant [47] (Table S2).

In the present work, only the free-run condition under constant light (LL) is simulated. Therefore, with L = 1, D = 0, and cP approaches zero after a long time of constant light [13-15], equations (1) to (3) can be further simplified as equations (6) to (8), in which we have omitted unnecessary parameters such as μ_{cca1} and μ_{cca2}.

$$\frac{d[cca]}{dt} = (\alpha_{cca1}.\beta_{cca} + \alpha_{cca2}.\beta_{cca}.Hill_{act\_lwd}).Hill_{rep\_prr}.Hill_{rep\_toc} - \gamma_{cca}.[cca], \quad (6)$$

$$\frac{d[prr]}{dt} = (\alpha_{prr1}.\beta_{prr} + \alpha_{prr2}.\beta_{prr}.Hill_{act\_lwd}).Hill_{act\_prr} - \gamma_{prr}.[prr], \quad (7)$$

$$\frac{d[toc]}{dt} = \beta_{toc}.Hill_{rep\_cca} - \gamma_{toc}.[toc]. \quad (8)$$

We reduced the parameter space by fixing the value of maximum possible steady-state concentration of each component to unity by assuming the Hill function as its maximum possible value, 1. In this way, the maximum production rates (β's) and total degradation rates (γ's) are set to be equal. The time t in the current work is in the unit of hours. After obtaining a regularly oscillating parameter set in the wild-type setting, we re-scaled all the time-related parameters such that the oscillation period is 24 hr.

b. Model M2

In model M2, equation (7) is modified as equation (9) for constant light,

$$\frac{d[prr]}{dt} = (\alpha_{prr1}.\beta_{prr} + \alpha_{prr2}.\beta_{prr}.Hill_{act\_lwd}).Hill_{rep\_toc} - \gamma_{prr}.[prr]. \quad (9)$$

whereas the other two equations remain the same (equations (6) and (8)). There is no additional parameter added in this new equation. The parameters were treated similarly as those in model M1.

c. Model M3 (Model A)

In model M3, equation (7) is further modified into equation (10),

$$\frac{d[prr]}{dt} = (\alpha_{prr1}.\beta_{prr} + \alpha_{prr2}.\beta_{prr}.Hill_{act\_lwd}).Hill_{rep\_toc}.Hill_{rep\_cca} - \gamma_{prr}.[prr]. \quad (10)$$

whereas the other two equations remain the same [equations (6) and (8)]. There is one additional parameter (the corresponding κ value in the new Hill function $Hill_{rep\_cca}$ for *PRR9/7*) added in this model. The parameters were treated similarly as those in model M1.

d. Model Pos-CCA

While for model Pos-CCA, equation (6) and (7) are modified into equation (11) and (12), respectively,

$$\frac{d[cca]}{dt} = (\alpha_{cca1}.\beta_{cca} + \alpha_{cca2}.\beta_{cca}.Hill_{act\_lwd}).Hill_{rep\_prr}.Hill_{rep\_toc}.Hill_{act\_cca} - \gamma_{cca}.[cca], \quad (11)$$

$$\frac{d[prr]}{dt} = (\alpha_{prr1}.\beta_{prr} + \alpha_{prr2}.\beta_{prr}.Hill_{act\_lwd}).Hill_{rep\_toc} - \gamma_{prr}.[prr]. \quad (12)$$

while equation (8) is kept the same. There is one additional parameter (the corresponding κ value in the new Hill function $Hill_{act\_cca}$ for *CCA1/LHY*) added in this model. The parameters were treated similarly as those in model M1.

e. Model M4 (Model B)

The model M4 (model M4-d, or model B in the main text) is described as a set of six ordinary differential equations (ODEs),

$$\frac{d[cca]}{dt} = \left(\alpha_{cca1}.\beta_{cca} + \alpha_{cca2}.\beta_{cca}.Hill_{act\_lwd}\right).Hill_{rep\_prr}.Hill_{rep\_toc} - \gamma_{cca}.[cca], \quad (13)$$

$$\frac{d[prr]}{dt} = \left(\alpha_{prr1}.\beta_{prr} + \alpha_{prr2}.\beta_{prr}.Hill_{act\_lwd}\right).Hill_{rep\_toc}.Hill_{rep\_cca} - \gamma_{prr}.[prr], \quad (14)$$

$$\frac{d[toc]}{dt} = \beta_{toc}.Hill_{rep\_cca}Hill_{rep\_EC} - \gamma_{toc}.[toc], \quad (15)$$

$$\frac{d[lux]}{dt} = \beta_{lux}.Hill_{rep\_cca}Hill_{rep\_EC}Hill_{rep\_toc} - \gamma_{lux}.[lux] - \beta_{EC}.[lux].[elf3], \quad (16)$$

$$\frac{d[elf3]}{dt} = \beta_{elf3}.Hill_{rep\_cca} - \gamma_{elf3}.[elf3] - \beta_{EC}.[lux].[elf3], \quad (17)$$

$$\frac{d[EC]}{dt} = -\beta_{EC}.[lux].[elf3] - \gamma_{EC}.[EC]. \quad (18)$$

Here, new components, [*lux*], [*elf3*], and [*EC*], denote a dimensionless concentration of *ELF4/LUX*, *ELF3*, and *Evening Complex (EC)*, respectively. There are 11 additional independent parameters added in model M4 (model M4-d), to facilitate the incorporation of *EC*. Finally, all parameters were treated similarly as described in model M1 above. For the other partial models (M4-a, M4-b, M4-c) we modified the equations for the removal of some interaction. For model M4-a, the equations (15) and (16) were modified into equations (19) and (20), respectively,

$$\frac{d[toc]}{dt} = \beta_{toc}.Hill_{rep\_cca} - \gamma_{toc}.[toc], \quad (19)$$

$$\frac{d[lux]}{dt} = \beta_{lux}.Hill_{rep\_cca}Hill_{rep\_EC} - \gamma_{lux}.[lux] - \beta_{EC}.[lux].[elf3]. \quad (20)$$

Next, for model M4-b, the equation (16) was modified into equation (20), while for model M4-c equation (15) was modified into equation (19) (as shown above). The other equations were kept the same as those for model M4 (model M4-d).

3. Supplementary results

a. Adding direct activation of *LWD1/2* to *PRR5/TOC1* reduced the performance of model M1

Previously, it has been shown that *LWD1/2* can bind to the promoter of *PRR5* and *TOC1*[28]. Unfortunately, we cannot find any noticeable improvement in adding this interaction into our model M1 (Fig. S1). We found that adding this interaction slightly increased the average hitting rate (Fig.

S1B). However, the performance of the genetic perturbation test was greatly reduced (Fig. S1C). This reduction is mostly caused by the inability of model M1_all in replicating the shorter period phenotype of *cca1/lhy* mutant test (Fig. S1C). Hence, we did not add this interaction in our current study because the supporting evidence of this interaction is still very limited. We thought some additional information regarding how *LWD1* modulates *PRR5* and *TOC1* expression is needed before we can properly model this interaction.

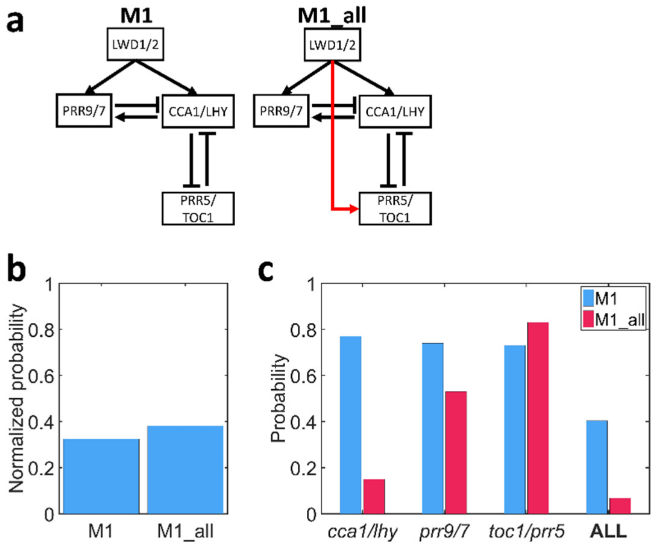

Figure S1. The comparison of the two different models.

(a) Schematic representation of the tested models.

(b) The averaged hitting rates of each parameters showing regular oscillation and correct *lwd1/2* mutant dynamics.

(c) The probability that the obtained parameter sets showed correct period changes in the genetic perturbation test results (*cca1/lhy*, *prr9/7*, *prr5/toc1*) for each model.

b. The direct inhibition of *CCA1/LHY* to *PRR9/7* is important for correct *prr9/7* genetic perturbation test result

Our findings showed that the changing of direct activation into indirect activation of *CCA1/LHY* to *PRR9/7* improved the robustness of the simplified models (Table S1). Unfortunately, the improved robustness is not followed by the improvement of model performance in replicating genetic perturbation test (Fig. S2A). Although the overall performance of M2 was greatly reduced, interestingly, it is mostly caused by the inability of model M2 in replicating *prr9/7* genetic perturbation test. On the other hand, the addition of a weak *CCA1/LHY* inhibition to *PRR9/7* can improve model A performance substantially (Fig.S2). These results imply that the direct inhibition of *CCA1/LHY* to *PRR9/7* might be important for the clock system, at least in reproducing *prr9/7* mutant condition.

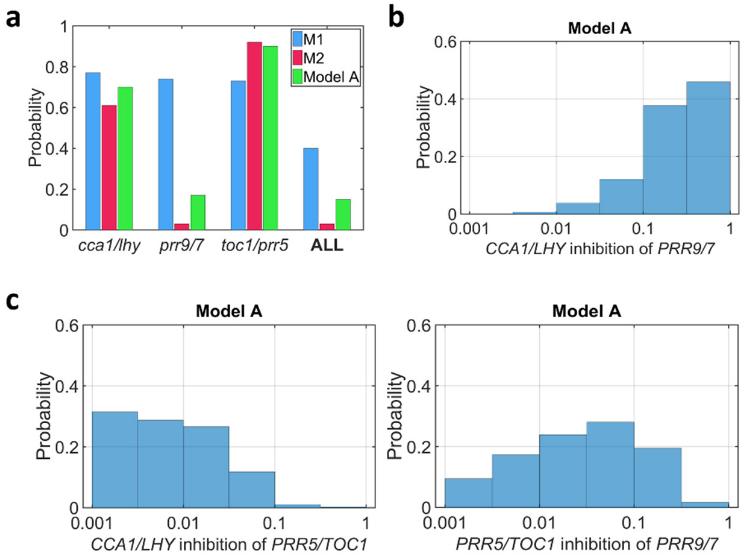

Figure S2. The addition of "weak" *CCA1/LHY* inhibition of *PRR9/7* improved the performance of model A.

(a) The probability that the obtained parameter sets showed a correct genetic perturbation test result (*cca1/lhy*, *prr9/7*, *prr5/toc1*) in each model.

(b) Parameter distribution of direct *CCA1/LHY* inhibition of *PRR9/7* in all selected parameter sets.

(c) Parameter distribution of indirect *CCA1/LHY* activation of *PRR9/7* via *PRR5/TOC1* in all selected parameter sets. (Left) Parameter distribution of *CCA1/LHY* inhibition of *PRR5/TOC1*. (Right) Parameter distribution of *PRR5/TOC1* inhibition of *PRR9/7*.

c. Under *CCA1/LHY* overexpression, the expression of *PRR9/7* was elevated in all models

In early study, with overexpression of *CCA1* and *LHY* gene, the expression of *PRR9* and *PRR7* was elevated [10]. Although model A has an indirect activation of *CCA1/LHY* to *PRR9/7*, whether it is also able to elevate the expression of *PRR9/7* gene under *CCA1/LHY* overexpression due to its direct inhibition is unclear. Hence, in this study, we tested whether all models can also replicate the observed experimental result. All models could replicate the elevated *PRR9/7* expression as observed in the experimental result (Fig. S3)

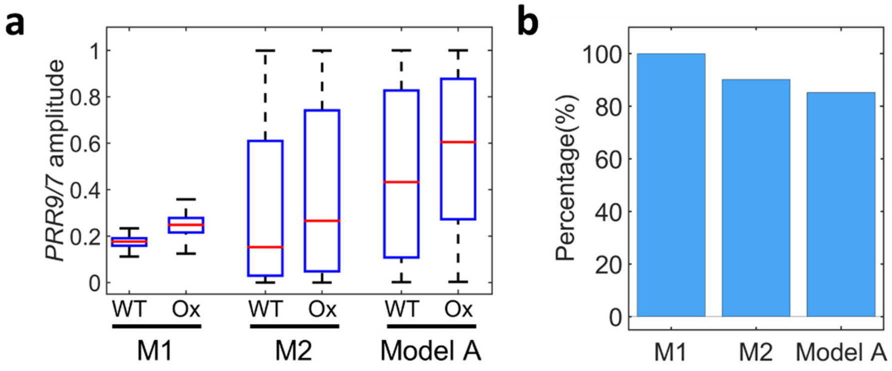

Figure S3. The increased amplitude of *PRR9/7* under *CCA1/LHY* overexpression is consistently seen in all models.

(a) Box plot showing the amplitude of *PRR9/7* in the wild type (WT) or consecutive *CCA1/LHY* overexpression condition (Ox) for all selected parameter sets. Red line indicates the median, and

box edges indicate the 25th (Q1) and 75th (Q3) percentiles. Whiskers are defined as 1.5*(Q3-Q1).

(b) Bar plot showing the percentage of parameter sets with increased amplitude under *CCA1/LHY* overexpression.

d. Only model model A shows an immediate reduction of *PRR9/7* expression under transient overexpression of *CCA1/LHY*

Recently, it was shown that the expression of *PRR9* and *PRR7* is actually reduced (instead of increased) in transient overexpression of *CCA1* and *LHY* [48,49]. Here, we did a transient overexpression by changing the initial condition value of *CCA1/LHY* gene, while keep the other parameters the same. Overtime, the system will eventually come back to their 'original' dynamics, while the immediate effects (initial changes) were correspondence to the effects of transient overexpression that has been seen in previous studies. Interestingly, we found that changing the initial condition of *CCA1/LHY* was actually shifting the phase of *PRR9/7* oscillation as shown in Fig. S4. Consequently, depending on the level of transient overexpression, we obtained a different concentration value of *PRR9/7* at a fixed time window. Therefore, to catch the overall behavior of many random parameter sets, we fixed the transient overexpression level and monitored the qualitative changes that was caused by the transient overexpression, rather than monitored *PRR9/7* expression at the fixed time window. As expected, only model A could replicate the reduced *PRR9/7* expression as well as an increase in the next *PRR9/7* amplitude.

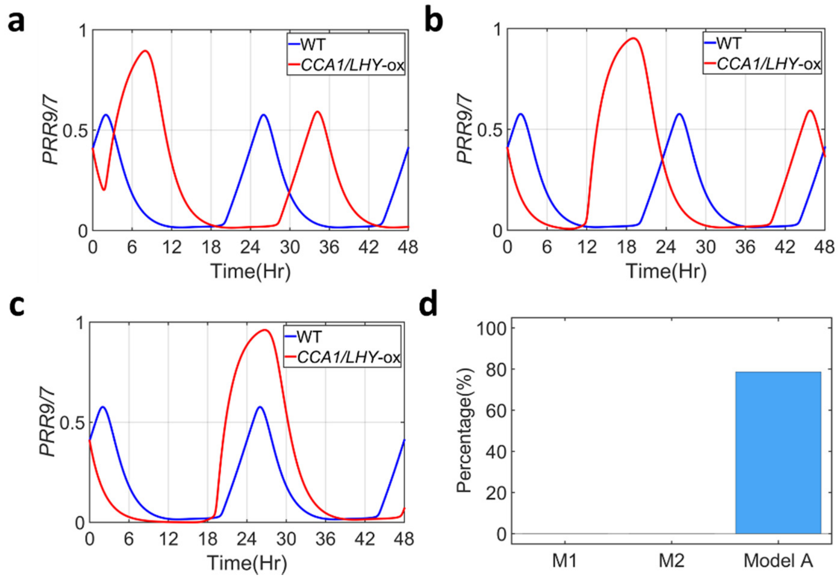

Figure S4. The expression of *PRR9/7* is shifted under transient *CCA1/LHY* overexpression.

(a-c) Comparison of *PRR9/7* expression under the wild type (blue line) or low (A), moderate (B), or high (C) *CCA1/LHY* overexpression (red line).

(d) Bar plot showing the number of parameter sets showing reduce *PRR9/7* expression (2 hr after induction) and an increase in the next *PRR9/7* amplitude (compared to the wild type).

e. PRR5/TOC1* generates pulse-like expression of *CCA1/LHY* gene and not *PRR9/7

In model A, *PRR5/TOC1* can act as an input for two different IFFL-2 pathways, which are going to *CCA1/LHY* and *PRR9/7*. Interestingly, as mentioned in the main text, only *CCA1/LHY* could generate a pulse-like behavior, when *PRR5/TOC1* turned off. Using this result, we were intrigued to understand the necessary condition for IFFL-2 in generating a pulse-like behavior in the output gene. Here we constructed a simple IFFL-2 model and scanned for a various plausible threshold values that can generate a pulse-like expression. As mentioned in the previous reports, IFFL acts as a sign-sensitive accelerator, such that, for IFFL-2, it can only create a pulse-like expression when the input gene is turning off [50]. Therefore, the input function (X) was set at high concentration initially, before it was turned off exponentially in our simulation (Fig. S5A, left). After that, the expression of target gene (Z) was monitored to detect the formation of any pulse at given parameter set values. Without loss of generality, we set the production and degradation value of gene Y and Z equal to one. Moreover, we found that the threshold value of Y repression to Z (κ_{zy}) was only contributed to the ability of the system to obtained a perfect adaptation as defined in previous study [51]. Therefore, for simplicity, we fixed the κ_{zy} value equal to 0.25 and focused our analysis on the effect of direct inhibition of input gene to the intermediate and output genes (Fig. S5A, left). Here, we found that, to generate a pulse-like expression, the inhibition of input gene to the output gene (κ_{zx}) must be weaker than its inhibition to the intermediate gene (κ_{yx}) (Fig. S5A, right). Intuitively, this constraint is needed because, when X turned off, a weaker κ_{zx} value will derepress first, which give Z enough time to accumulate before it is finally repressed by the intermediate gene Y. Thus, with this insight, we compared the threshold value of *PRR5/TOC1* inhibition to both *CCA1/LHY* (κ_{CT}) and *PRR9/7* (κ_{PT}) (Fig. S5B). The result shows that, in most of the parameter sets, the κ_{CT} were always had lower value compared to κ_{PT}, which explained why we could only observed a pulse-like expression of *CCA1/LHY* gene, and not *PRR9/7* (Fig. S5B, right).

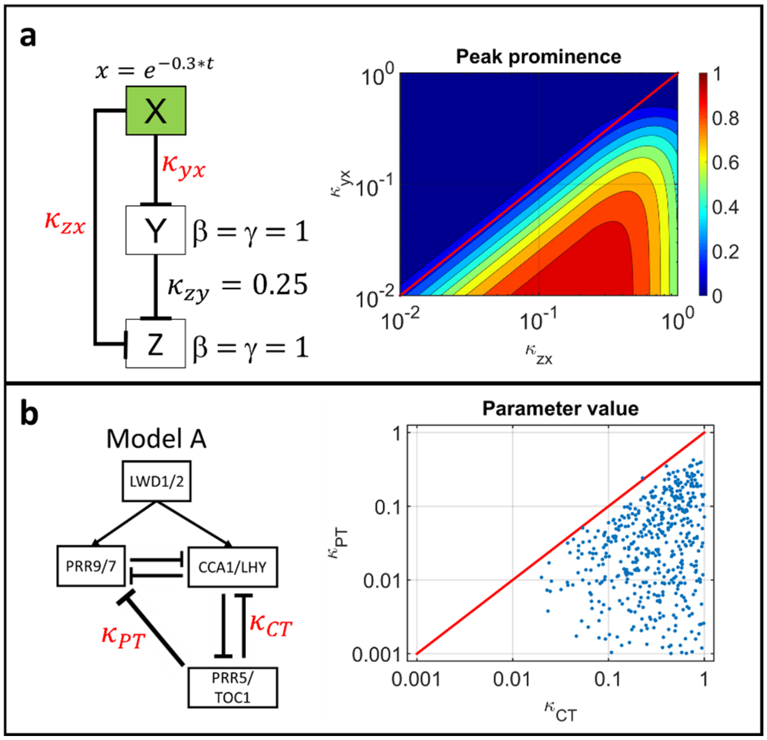

Figure S5. Weaker inhibition of input gene to output gene is needed for creating pulse-like expression of the output gene in IFFL-2.

(a) The distribution of two parameter values (κ_{zx} and κ_{yx}) that can produce a pulse-like expression in a simple IFFL-2 network. Genes in the green box are treated as an input function during the simulation. Red line indicates κ_{zx} equal to κ_{yx}.

(b) The distribution of *PRR5/TOC1* inhibition to *CCA1/LHY* (κ_{CT}) compared with *PRR5/TOC1* inhibition to *PRR9/7* (κ_{PT}) in model A. Red line indicates κ_{CT} equal to κ_{PT}.

f. The IFFL-2 from *CCA1/LHY* to *PRR9/7* helps increase *CCA1/LHY* expression level

We first found that models M1, M2 and model A had zero, one, and two IFFL-2s in the network, respectively. Surprisingly, the increased number of IFFL-2s was associated with the distribution of *CCA1/LHY* amplitude in each model (Fig. S6). Later on, as discussed in the main text, the IFFL-2 from *CCA1/LHY* to *PRR9/7* in model A was able to delay the expression of *PRR9/7*, which allowed *CCA1/LHY* to accumulate longer. Thus, the ability of IFFL-2 in delaying the expression of output genes might be important for the clock system.

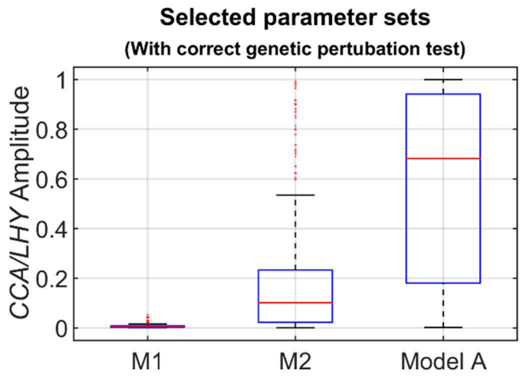

Figure S6. The distribution of *CCA1/LHY* amplitude in all parameter sets showing correct genetic perturbation test results. Red line indicates the median, and box edges indicate the 25th (Q1) and 75th (Q3) percentiles. Whiskers are defined as 1.5*(Q3-Q1).

g. Different *EC* representation has little impact on model performance and dynamics

The knowledge of how *EC* is regulated and how it works in the clock system is still limited, as shown by the discrepancy in many mathematical models in describing *EC*. For example, in P2012, the *EC* was described as a repressor of *PRR9*, *TOC1*, and *GI*, while in turn being regulated by *CCA1/LHY*, and *COP1* [15]. However, in F2014, the *EC* was described as "rate-limited by *LUX* and *NOX* on one hand and by *ELF3-ELF4* and free *ELF3* on the other" [46]. There, *EC* repressed seven target genes — *PRR9*, *PRR7*, *PRR5*, *TOC1*, *LUX*, *ELF4,* and *GI* — whereas *CCA1* and *LHY* genes inhibited *ELF3*, *ELF4*, *LUX,* and *NOX*, and *TOC1* inhibited *ELF4* and *LUX* [46]. Moreover, recently, Caluwe *et al.* (C2016) built a compact model showing *EC* inhibited by *CCA1/LHY* and itself (auto-negative feedback) but conversely repressing the expression of *PRR9/7*.

Therefore, in this study, we built four different models to test the effect of different interactions of EC (Fig. S7A). Among 9,000,000 random parameter sets searched in this study (Table S1), they had a comparable hitting rate, or the difference was unnoticeable (Fig. S7B). A similar result was also found in the genetic perturbation test, in which their performance was also comparable (Fig. S7C). These results indicate that the difference in EC regulation had little impact on the dynamics of the system. Thus, we used model M4-d (Model B in the main text) to accommodate all observed experimental results.

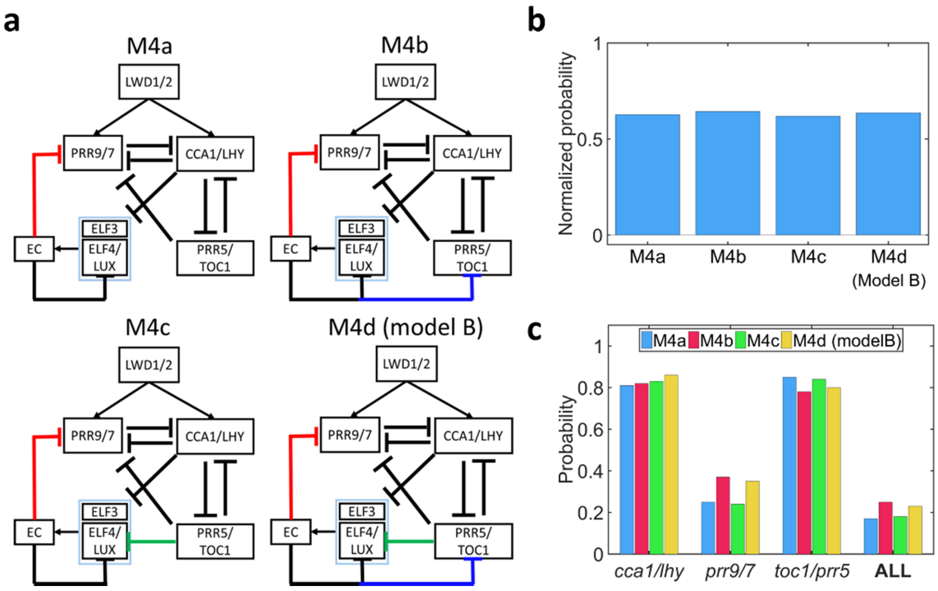

Figure S7. The comparison of different *EC* interactions.

(a) Schematic representation of the tested interaction.

(b) The averaged hitting rates of each parameter showing regular oscillation and correct *lwd1/2* mutant dynamics.

(c) The probability that the obtained parameter set showed correct genetic perturbation test results (*cca1/lhy*, *prr9/7*, *prr5/toc1*) for each model.

h. The insights we obtained from model A can also be seen in model B

After adding EC into model A, the next question was whether the previous insights were still maintained in model B. As we discuss in the main text, we first tested whether the *CCA1/LHY-PRR5/TOC1* double-negative feedback was still able to show bistability under *PRR9/7* titration. Here, we found that 98.41% of parameter sets were still able to generate bistability in model B (Fig. S8). In addition, 66.67% of parameter sets could also show bistability under EC titration, but had the opposite effect as compared with *PRR9/7* titration (main text) (Fig. S8). Therefore, these results suggest that the bistability found in the *CCA1/LHY-PRR5/TOC1* double-negative feedback loop is still maintained in the new, more complex, model B.

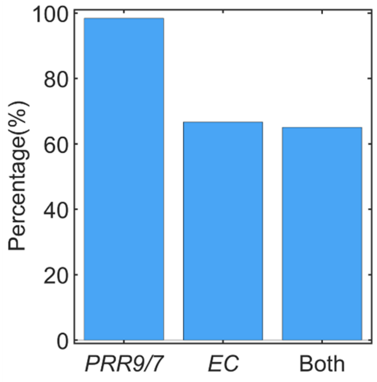

Figure S8. Bar plot showing the number of parameter sets with bistability under the titration of *PRR9/7*, *EC*, and both.

Next, we again tested the ability of IFFL-2 to generate a pulse-like expression of *PRR9/7* in model B. As discussed in the main text, *PRR9/7* still showed pulse-like expression, as shown in Fig. S9A. However, *EC* did not show pulse-like expression as observed in *PRR9/7* (Fig. S9B). This result drew our attention because one of *EC* subunits, *ELF4/LUX*, was actually under two different sources of IFFL-2 (one from *CCA1/LHY* and the other from *PRR5/TOC1*). However, in addition to the two IFFL-2 regulations, *ELF4/LUX* was also under a regulation of an auto-negative feedback loop. Thus, we tried to understand why, despite having two IFFL-2s regulating its expression, *EC* did not show a more pulse-like expression in model B. To answer this question, we constructed a simple IFFL-2 model but added an auto-negative feedback loop in the output gene to mimic the *ELF4/LUX* regulation (Fig. S10 left). Similar to previous analysis, we fixed some of the parameter values and focused our analysis on the effect of different threshold values of input inhibition of the intermediate and output genes (Fig. S10 left). Similar to previous results, the pulse-like expression was only seen when the X inhibition of Z (κ_{zx}) was weaker than the X inhibition of Y (κ_{yx}) (Fig. S10 right). However, the peak prominence was reduced significantly as compared with the previous model, which had no auto-negative feedback loop (compare Fig. S10 with Fig. S5A). These results implied that a combination of IFFL-2 and auto-negative feedback loop might reduce the ability of the output gene to generate a pulse-like expression, which might explain in part why *EC* cannot generate a more pulse-like expression despite being regulated by two different IFFL-2.

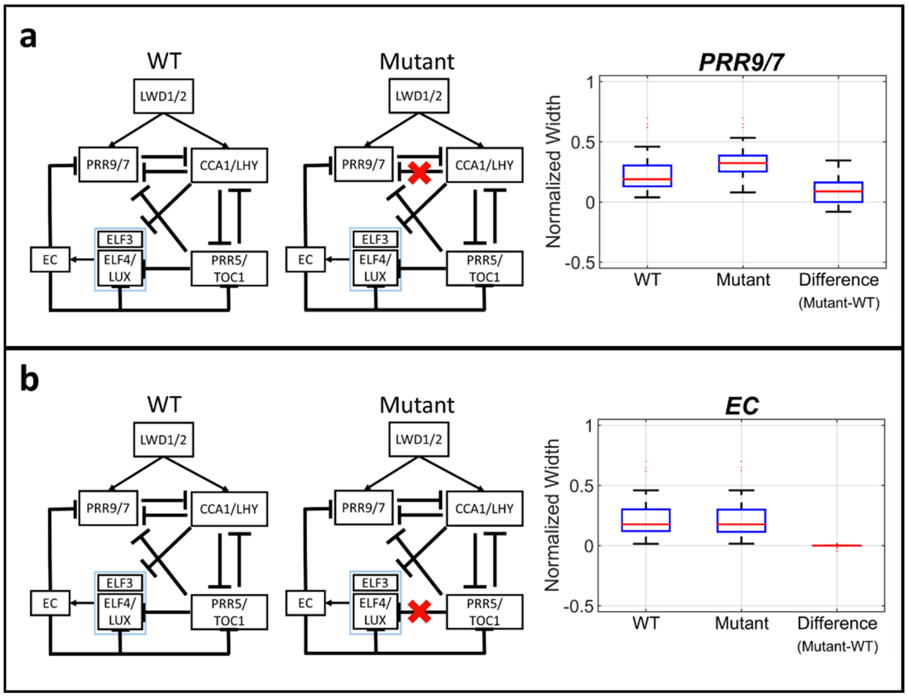

Figure S9. The pulse-like expression was seen only in *PRR9/7* and not *EC*.

(a) Box plot representing the normalized width of *PRR9/7* gene for all selected parameter sets in both WT and mutant condition.

(b) Box plot representing the normalized width of *EC* gene for all selected parameter sets in both WT and mutant condition.

Red line indicates the median, and box edges indicate the 25th (Q1) and 75th (Q3) percentiles. Whiskers are defined as 1.5*(Q3-Q1). The mutation involved multiplying the selected threshold parameter with a very large number (1×10^9).

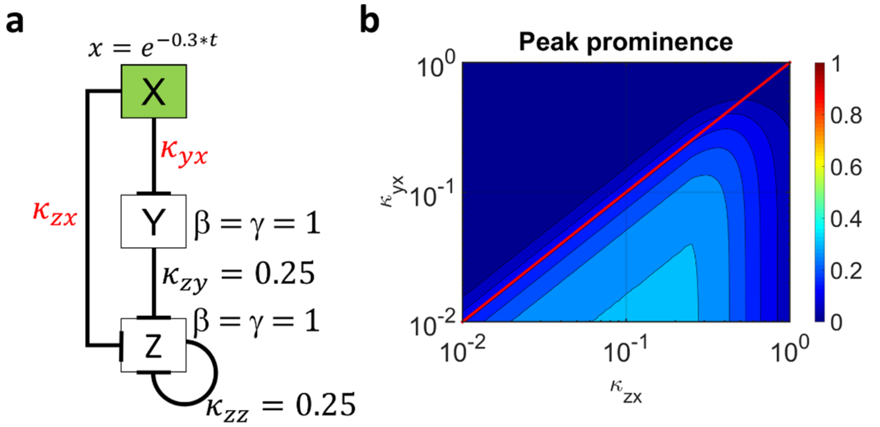

Figure S10. Adding auto-negative feedback loop reduced the ability of IFFL-2 to generate a pulse-like expression.

(a) Schematic representation of the simple IFFL-2 network with additional auto-negative feedback loop. Genes in the green box are treated as an input function during the simulation.

(b) The distribution of two parameter values (κ_{zx} and κ_{yx}) that can produce a pulse-like expression in a simple IFFL-2 network with an additional auto-negative feedback loop. Red line indicates κ_{zx} equal to κ_{yx}.

i. Our findings are general and are not limited to certain model structures

As we discussed previously in the main text, there are limitations for a simple model. For instance, in order to reduce the model complexity, we have to compromise and skip some of the detailed interactions as well as the precise expression profiles. Therefore, we need to show that the insights we obtained using simple model remains in the more detailed or complex model. Here, we tested the insights we obtained using two different detailed model P2012 [15] and F2014 [46]. These two models include many known clock genes, can replicate multiple genetic perturbation observed in the experimental result, and represented a different presentation of describing *PRRs* and *EC* gene inside the clock systems [15,46]. Furthermore, P2012 has also been extended to simulate and interpret the input [37] and output [38] pathways.

At first, we tested whether the double negative feedback loop between *CCA1/LHY* and *TOC1* could also generate a bistable, hysteresis behavior in both models. Here, we performed a similar analysis as we did before for model A and model B (methods). Interestingly, we found that both models were still able to generate a bistable, hysteretic behavior under different *PRR9* concentration (Fig. S11). This result is intriguing remembering both models have different representation of how *CCA1/LHY* and *PRR9* genes are regulated. Thus, we would like to argue that the bistability under different *PRR9* concentration might be essential to the clock system, such that it is retrained in many models regardless how *CCA1/LHY* and *PRRs* genes are regulated.

On the other hand, we found that only model F2014 could show a bistable, hysteretic behavior under different EC concentration (Fig. S11). In the P2012 model, the system still oscillated mildly under

certain constant *EC* concentration. This mild oscillation prohibited us to obtain the steady state concentration of both *CCA1/LHY* and *TOC1* in the P2012 model. Hence, the results of model P2012 cannot be compared with other model for this analysis. However, the fact that we can still observe a bistability in model F2014 is still interesting, because *EC* was describe differently in F2014 and our simplified models. Therefore, we would like to argue that the ability of *EC* in generating a bistability is still general and not an artifact of the simplification process.

Lastly, we also tested whether the *PRR9* and *PRR7* genes showed a more pulse-like behavior in the other clock models. Unfortunately, in P2012, the *PRR9* and *PRR7* are still under direct activation of *CCA1/LHY* gene and do not contain a similar IFFL-2 that we tested previously using our simplified model. Hence, we cannot performed a similar analysis using P2012 and would only perform it on F2014. Here, we found that only *PRR7* showed a more pulse-like expression in model F2014 and not *PRR9* (Fig. S12). This result might happen because, as stated in the original paper, the *PRR9* was virtually unaffected to *CCA1* and *LHY* repression or having a weak repression in F2014 model [46]. For the *PRR7* gene, we found that, while the period of the system was not altered, the expression of *PRR7* was significantly elevated. Hence, it made *PRR7* to express longer and less pulse-like, as we seen in our simplified model. Thus, this result supports our argument that the more pulse-like expression of PRRs genes is not an artifact of our simplification process.

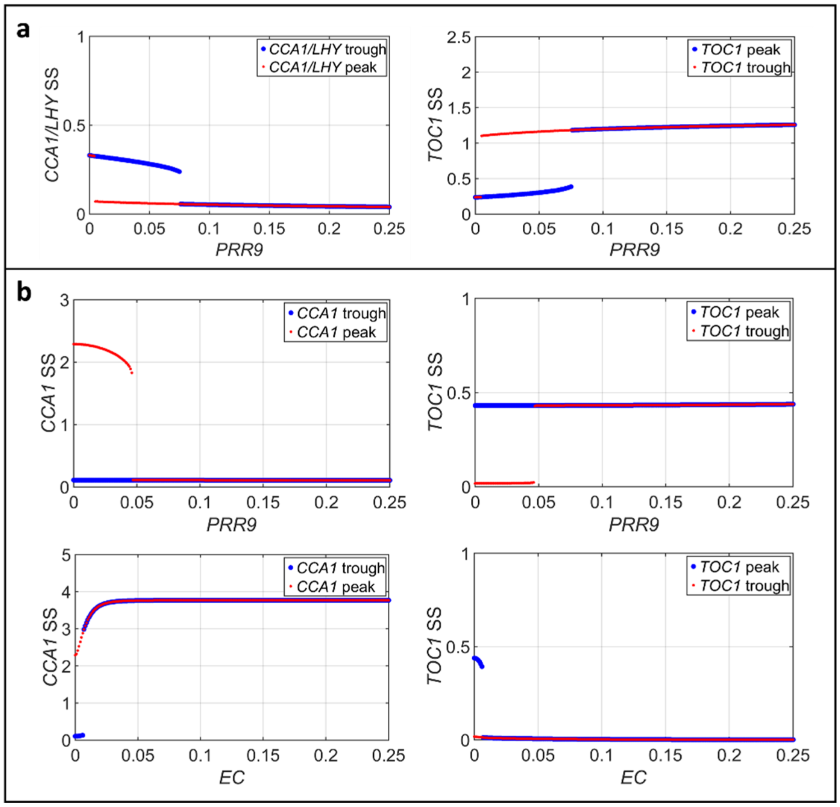

Figure S11. Previous mathematical models also show a bistable, hysteretic behavior as observed in our simplified models

(a) Model P2012.
(b) Model F2014.

The expression of *CCA1/LHY* (left) and *TOC1* (right) also show a hysteretic switch bistability under different *PRR9* concentrations. However, under *EC* titration, the hysteresis is only seen in model F2014 (B, below). Dots represent the steady state (SS) value of *CCA1/LHY* or *TOC1* level starting from a low level of *CCA1/LHY* / high level of *TOC1* (blue dots) or high level of *CCA1/LHY* / low level of *TOC1* (red dots).

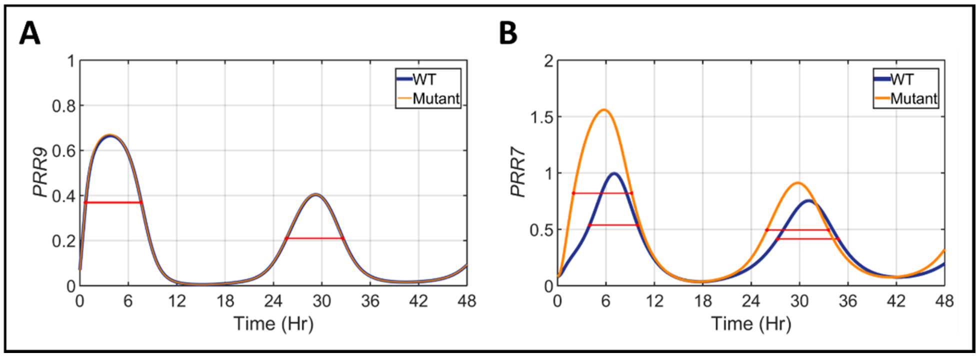

Figure S12. Only *PRR7* (and not *PRR9*) shows pulse-like expression in model F2014.
(a) The expression of *PRR9* under the WT (blue) or mutant condition (orange).
(b) The expression of *PRR7* under the WT (blue) or mutant condition (orange).
The mutant condition involved multiplying the threshold parameter of *CCA1* and *LHY* inhibition to *PRR9* (A) or *PRR7* (B) with a very large number ($1x10^9$). The red line represents the full width at half maximum (FWHM) for each gene.

j. The comparison of real experimental data with the simulated result found in model A

We further tested the performance of our simplified model by comparing the expression profile of all the genes presented in model A to the experimental data obtained from the literature[28] (Fig S13). To simplify the comparison process, we normalized all data, both experimental and simulation, to their maximum values. Moreover, we also removed the linear trend found in expression of *PRR5* and *TOC1* (Fig S13A). The results shown that both *CCA1/LHY* and *PRR9/7* expression profile were comparable with the experimental results (Fig S13 B and C). However, the phase of *PRR5/TOC1* in our simulation was slightly delayed compared to the experimental result (Fig S13D). Interestingly, such a delay is also seen in the previous simplified model C2016[33]. We speculate that this delayed phase might happen because, in both simplified models, they were lacked of *PRR5* and *TOC1* activator, *RVE8*, as well as the protein degradator, ZTL.

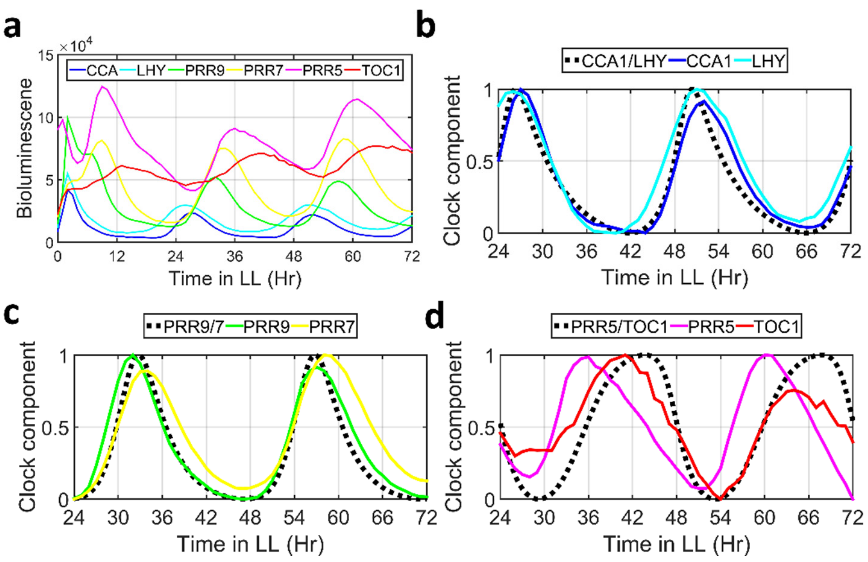

Figure S13. The comparison of experimental and simulated experimental profile.

(a) The bioluminescence data of *CCA1*, *LHY*, *PRR9*, *PRR9*, *PRR5*, and *TOC1* in the wild type condition.
(b) The comparison of simulated *CCA1/LHY* with the experimental result.
(c) The comparison of simulated *PRR9/7* with the experimental result.
(d) The comparison of simulated *PRR5/TOC1* with the experimental result.

All data present in the panel (b-d) was normalized to their maximum value. The simulated data were presented in the black-dashed curve. The experimental data was obtained from Wang *et al.* 2011[28].

4. Supplementary Tables

a. Table S1. Probability of finding proper parameter sets of all models

Model	# of independent parameter	# parameter searched	# parameter obtained	Probability[a]	Average probability
M1	12	240,000,000	1,005	4.19 x10^{-06}	0.3244
M2	12	9,000,000	11,073	1.23 x10^{-03}	0.5721
M3[b]	13	9,000,000	3,113	3.46 x10^{-04}	0.5417
M4-a	21	9,000,000	499	5.54 x10^{-05}	0.6271
M4-b	22	9,000,000	546	6.07 x10^{-04}	0.6432
M4-c	22	9,000,000	233	2.59 x10^{-05}	0.6187
M4-d[b]	23	9,000,000	277	3.08 x10^{-05}	0.6366

[a] Probability of passing our criteria of producing sustained oscillation and proper *lwd1/2* mutant behavior.
[b] M3 is model A and M4-d is model B in the main text.

b. Table S2. Search ranges for parameters

Parameters	Range	Units	Search scale
γ's [1]	0.01–10	1/Hr	Logarithm
α_{prr1}, α_{cca1} [2]	0–1	Dimensionless	Linear
κ's (in all Hill functions)	0.001–1	Dimensionless	Logarithm
n's (in all Hill function) [3]	2–8	Dimensionless	Linear

[1] β's are set to the same value as the corresponding γ's for a dimensionless unit for the concentration of each gene and for a reduction in number of parameters.
[2] Without loss of generality, we set $\alpha_{prr2} = 1 - \alpha_{prr1}$ and $\alpha_{cca2} = 1 - \alpha_{cca1}$ (for all models).
[3] Only integer value was used for this hill coefficient.

5. Supplementary References

1 Hsu, P. Y. & Harmer, S. L. Wheels within wheels: the plant circadian system. *Trends in plant science* **19**, 240-249, doi:10.1016/j.tplants.2013.11.007 (2014).

2 Mizoguchi, T. *et al.* LHY and CCA1 are partially redundant genes required to maintain circadian rhythms in Arabidopsis. *Dev Cell* **2**, 629-641 (2002).

3 Nakamichi, N. *et al.* PSEUDO-RESPONSE REGULATORS 9, 7, and 5 are transcriptional repressors in the Arabidopsis circadian clock. *Plant Cell* **22**, 594-605, doi:10.1105/tpc.109.072892 (2010).

4 Gendron, J. M. *et al.* Arabidopsis circadian clock protein, TOC1, is a DNA-binding transcription factor. *Proceedings of the National Academy of Sciences of the United States of America* **109**, 3167-3172, doi:10.1073/pnas.1200355109 (2012).

5 Huang, W. *et al.* Mapping the core of the Arabidopsis circadian clock defines the network structure of the oscillator. *Science* **336**, 75-79, doi:10.1126/science.1219075 (2012).

6 Alabadi, D. *et al.* Reciprocal regulation between TOC1 and LHY/CCA1 within the Arabidopsis circadian clock. *Science* **293**, 880-883, doi:10.1126/science.1061320 (2001).

7 Kikis, E. A., Khanna, R. & Quail, P. H. ELF4 is a phytochrome-regulated component of a negative-feedback loop involving the central oscillator components CCA1 and LHY. *The Plant journal : for cell and molecular biology* **44**, 300-313, doi:10.1111/j.1365-313X.2005.02531.x (2005).

8 Hazen, S. P. *et al.* LUX ARRHYTHMO encodes a Myb domain protein essential for circadian rhythms. *Proceedings of the National Academy of Sciences of the United States of America* **102**, 10387-10392, doi:10.1073/pnas.0503029102 (2005).

9 Helfer, A. *et al.* LUX ARRHYTHMO encodes a nighttime repressor of circadian gene expression in the Arabidopsis core clock. *Current biology : CB* **21**, 126-133, doi:10.1016/j.cub.2010.12.021 (2011).

10 Farre, E. M., Harmer, S. L., Harmon, F. G., Yanovsky, M. J. & Kay, S. A. Overlapping and distinct roles of PRR7 and PRR9 in the Arabidopsis circadian clock. *Current biology : CB* **15**, 47-54, doi:10.1016/j.cub.2004.12.067 (2005).

11 Kawamura, M., Ito, S., Nakamichi, N., Yamashino, T. & Mizuno, T. The function of the clock-associated transcriptional regulator CCA1 (CIRCADIAN CLOCK-ASSOCIATED 1) in Arabidopsis thaliana. *Bioscience, biotechnology, and biochemistry* **72**, 1307-1316, doi:10.1271/bbb.70804 (2008).

12 Bujdoso, N. & Davis, S. J. Mathematical modeling of an oscillating gene circuit to unravel the circadian clock network of Arabidopsis thaliana. *Front Plant Sci* **4**, 3, doi:10.3389/fpls.2013.00003 (2013).

13 Locke, J. C. *et al.* Extension of a genetic network model by iterative experimentation and mathematical analysis. *Mol Syst Biol* **1**, 2005 0013, doi:10.1038/msb4100018 (2005).

14 Pokhilko, A. *et al.* Data assimilation constrains new connections and components in a complex, eukaryotic circadian clock model. *Mol Syst Biol* **6**, 416, doi:10.1038/msb.2010.69 (2010).

15 Pokhilko, A. *et al.* The clock gene circuit in Arabidopsis includes a repressilator with additional feedback loops. *Mol Syst Biol* **8**, 574, doi:10.1038/msb.2012.6 (2012).

16 Matsushika, A., Makino, S., Kojima, M. & Mizuno, T. Circadian waves of expression of the APRR1/TOC1 family of pseudo-response regulators in Arabidopsis thaliana: insight into the plant circadian clock. *Plant & cell physiology* **41**, 1002-1012 (2000).

17 Somers, D. E., Webb, A. A., Pearson, M. & Kay, S. A. The short-period mutant, toc1-1, alters circadian clock regulation of multiple outputs throughout development in Arabidopsis thaliana. *Development (Cambridge, England)* **125**, 485-494 (1998).

18 Bell-Pedersen, D. *et al.* Circadian rhythms from multiple oscillators: lessons from diverse organisms. *Nature reviews. Genetics* **6**, 544-556, doi:10.1038/nrg1633 (2005).

19 Para, A. *et al.* PRR3 Is a Vascular Regulator of TOC1 Stability in the Arabidopsis Circadian Clock. *The Plant Cell* **19**, 3462-3473, doi:10.1105/tpc.107.054775 (2007).

20 Copenhaver, G. P. *et al.* REVEILLE8 and PSEUDO-REPONSE REGULATOR5 Form a Negative Feedback Loop within the Arabidopsis Circadian Clock. *PLoS Genetics* **7**, e1001350, doi:10.1371/journal.pgen.1001350 (2011).

21 Hsu, P. Y., Devisetty, U. K. & Harmer, S. L. Accurate timekeeping is controlled by a cycling activator in Arabidopsis. *eLife* **2**, e00473, doi:10.7554/eLife.00473 (2013).

22 Wu, J. F., Wang, Y. & Wu, S. H. Two new clock proteins, LWD1 and LWD2, regulate Arabidopsis photoperiodic flowering. *Plant Physiol* **148**, 948-959, doi:10.1104/pp.108.124917 (2008).

23 Mas, P., Kim, W. Y., Somers, D. E. & Kay, S. A. Targeted degradation of TOC1 by ZTL modulates circadian function in Arabidopsis thaliana. *Nature* **426**, 567-570, doi:10.1038/nature02163 (2003).
24 Kiba, T., Henriques, R., Sakakibara, H. & Chua, N. H. Targeted degradation of PSEUDO-RESPONSE REGULATOR5 by an SCFZTL complex regulates clock function and photomorphogenesis in Arabidopsis thaliana. *Plant Cell* **19**, 2516-2530, doi:10.1105/tpc.107.053033 (2007).
25 Wang, L., Fujiwara, S. & Somers, D. E. PRR5 regulates phosphorylation, nuclear import and subnuclear localization of TOC1 in the Arabidopsis circadian clock. *The EMBO journal* **29**, 1903-1915, doi:10.1038/emboj.2010.76 (2010).
26 Yamamoto, Y. *et al.* Comparative genetic studies on the APRR5 and APRR7 genes belonging to the APRR1/TOC1 quintet implicated in circadian rhythm, control of flowering time, and early photomorphogenesis. *Plant & cell physiology* **44**, 1119-1130 (2003).
27 Michael, T. P. *et al.* Enhanced fitness conferred by naturally occurring variation in the circadian clock. *Science* **302**, 1049-1053, doi:10.1126/science.1082971 (2003).
28 Wang, Y. *et al.* LIGHT-REGULATED WD1 and PSEUDO-RESPONSE REGULATOR9 form a positive feedback regulatory loop in the Arabidopsis circadian clock. *Plant Cell* **23**, 486-498, doi:10.1105/tpc.110.081661 (2011).
29 Farre, E. M. & Kay, S. A. PRR7 protein levels are regulated by light and the circadian clock in Arabidopsis. *The Plant journal : for cell and molecular biology* **52**, 548-560, doi:10.1111/j.1365-313X.2007.03258.x (2007).
30 Ito, S., Nakamichi, N., Kiba, T., Yamashino, T. & Mizuno, T. Rhythmic and light-inducible appearance of clock-associated pseudo-response regulator protein PRR9 through programmed degradation in the dark in Arabidopsis thaliana. *Plant Cell Physiol* **48**, 1644-1651, doi:10.1093/pcp/pcm122 (2007).
31 Nakamichi, N., Kita, M., Ito, S., Yamashino, T. & Mizuno, T. PSEUDO-RESPONSE REGULATORS, PRR9, PRR7 and PRR5, together play essential roles close to the circadian clock of Arabidopsis thaliana. *Plant & cell physiology* **46**, 686-698, doi:10.1093/pcp/pci086 (2005).
32 Kim, W. Y. *et al.* ZEITLUPE is a circadian photoreceptor stabilized by GIGANTEA in blue light. *Nature* **449**, 356-360, doi:10.1038/nature06132 (2007).
33 De Caluwé, J. *et al.* A Compact Model for the Complex Plant Circadian Clock. *Frontiers in Plant Science* **7**, 74, doi:10.3389/fpls.2016.00074 (2016).
34 Yu, J. W. *et al.* COP1 and ELF3 control circadian function and photoperiodic flowering by regulating GI stability. *Mol Cell* **32**, 617-630, doi:10.1016/j.molcel.2008.09.026 (2008).
35 Xie, Q. *et al.* LNK1 and LNK2 are transcriptional coactivators in the Arabidopsis circadian oscillator. *Plant Cell* **26**, 2843-2857, doi:10.1105/tpc.114.126573 (2014).
36 Locke, J. C. *et al.* Experimental validation of a predicted feedback loop in the multi-oscillator clock of Arabidopsis thaliana. *Mol Syst Biol* **2**, 59, doi:10.1038/msb4100102 (2006).
37 Gould, P. D. *et al.* Network balance via CRY signalling controls the Arabidopsis circadian clock over ambient temperatures. *Molecular Systems Biology* **9**, doi:10.1038/msb.2013.7 (2013).
38 Seaton, D. D. *et al.* Linked circadian outputs control elongation growth and flowering in response to photoperiod and temperature. *Mol Syst Biol* **11**, 776, doi:10.15252/msb.20145766 (2015).
39 Wu, J.-F. *et al.* LWD–TCP complex activates the morning gene CCA1 in Arabidopsis. *Nature Communications* **7**, 13181, doi:10.1038/ncomms13181 (2016).
40 Kim, J. Y., Song, H. R., Taylor, B. L. & Carre, I. A. Light-regulated translation mediates gated induction of the Arabidopsis clock protein LHY. *The EMBO journal* **22**, 935-944, doi:10.1093/emboj/cdg075 (2003).
41 Ito, S. *et al.* Molecular dissection of the promoter of the light-induced and circadian-controlled APRR9 gene encoding a clock-associated component of Arabidopsis thaliana. *Bioscience, biotechnology, and biochemistry* **69**, 382-390, doi:10.1271/bbb.69.382 (2005).
42 Alon, U. *An Introduction to Systems Biology: Design Principles of Biological Circuits*. (Taylor & Francis, 2006).
43 Ferrell, J. E., Jr. & Ha, S. H. Ultrasensitivity part II: multisite phosphorylation, stoichiometric inhibitors, and positive feedback. *Trends in biochemical sciences* **39**, 556-569, doi:10.1016/j.tibs.2014.09.003 (2014).
44 Fujiwara, S. *et al.* Post-translational regulation of the Arabidopsis circadian clock through selective proteolysis and phosphorylation of pseudo-response regulator proteins. *The Journal of biological chemistry* **283**, 23073-23083, doi:10.1074/jbc.M803471200 (2008).
45 Yakir, E. *et al.* Posttranslational regulation of CIRCADIAN CLOCK ASSOCIATED1 in the circadian oscillator of Arabidopsis. *Plant Physiol* **150**, 844-857, doi:10.1104/pp.109.137414 (2009).

46 Fogelmark, K. & Troein, C. Rethinking Transcriptional Activation in the <italic>Arabidopsis</italic> Circadian Clock. *PLoS Comput Biol* **10**, e1003705, doi:10.1371/journal.pcbi.1003705 (2014).

47 Nagel, D. H. & Kay, S. A. Complexity in the wiring and regulation of plant circadian networks. *Current biology : CB* **22**, R648-657, doi:10.1016/j.cub.2012.07.025 (2012).

48 Adams, S., Manfield, I., Stockley, P. & Carré, I. A. Revised Morning Loops of the <italic>Arabidopsis</italic> Circadian Clock Based on Analyses of Direct Regulatory Interactions. *PLoS ONE* **10**, e0143943, doi:10.1371/journal.pone.0143943 (2015).

49 Kamioka, M. *et al.* Direct Repression of Evening Genes by CIRCADIAN CLOCK-ASSOCIATED1 in the Arabidopsis Circadian Clock. *Plant Cell* **28**, 696-711, doi:10.1105/tpc.15.00737 (2016).

50 Mangan, S. & Alon, U. Structure and function of the feed-forward loop network motif. *Proceedings of the National Academy of Sciences* **100**, 11980-11985, doi:10.1073/pnas.2133841100 (2003).

51 Ma, W., Trusina, A., El-Samad, H., Lim, W. A. & Tang, C. Defining Network Topologies that Can Achieve Biochemical Adaptation. *Cell* **138**, 760-773, doi:http://dx.doi.org/10.1016/j.cell.2009.06.013 (2009).
