# Supplementary material for: An incoherent feed-forward loop switches the Arabidopsis clock rapidly between two hysteretic states
Source: Sci Rep. 2018 Sep 17;8:13944. doi: 10.1038/s41598-018-32030-z (PMC6141573; doi:10.1038/s41598-018-32030-z)
Supplement: Supplementary file 2 — Supplementary dataset [file 41598_2018_32030_MOESM2_ESM.zip › Code/Readme.pdf]

Usage of the codes:

1) Run the r1_searching_octave.m to obtain random parameter sets that is oscillating, has shorter period and lower period under *lwd1/2* mutant condition. (It is written in octave language)
2) The r2_genpertub_test.m is used to perform the genetic perturbation test for each obtain parameter sets in previous steps.
3) The r3_data_prepare.m is for preparing results obtained in step 2 to be used in step 4.
4) The r4_genpertub_select.m is used to select parameter sets that have correct genetic perturbation test (shorter period in *cca1/lhy*, longer period in *prr9/7*, and shorter period in *prr5/toc1* mutant conditions).

We also provide our final results named as …_param_selected.csv. This result was obtained after step 4. The parameter sets is used for further analysis in our study.

The functions folder contains necessary script to perform the searching and selection process.
